# Sex- and ALDH2-dependent differences in alcohol metabolism and psychomotor performance: a study in Han Chinese adults after binge drinking

**DOI:** 10.1080/07853890.2025.2496798

**Published:** 2025-04-28

**Authors:** Yi Ye, Yidie Lin, Takeshi Haseba, Fan Chen, Fanlai Cui, Xiaoqin Yi, Weihao Fan, Gangqin Li

**Affiliations:** ^a^ Department of Forensic Toxicological Analysis, West China School of Basic Medical Sciences & Forensic Medicine, Sichuan University, Chengdu, China; ^b^ Department of Epidemiology and Health Statistics, West China School of Public Health and West China Fourth Hospital, Sichuan University, Chengdu, China; ^c^ Department of Forensic Medicine, Kanagawa Dental University, Yokosuka, Japan; ^d^ Department of Legal Medicine, Nippon Medical School, Tokyo, Japan; ^e^ School of Basic Medical Sciences and Forensic Medicine, Hangzhou Medical College, Hangzhou, China; ^f^ Department of Forensic Psychiatry, West China School of Basic Medical Sciences & Forensic Medicine, Sichuan University, Chengdu, China

**Keywords:** Alcohol metabolism, psychomotor dysfunction, *ALDH2* polymorphism, sex, alcohol consumption

## Abstract

**Background:**

Psychomotor impairments due to alcohol consumption may lead to a series of negative consequences. However, the influence of sex and *ALDH2* polymorphism on psychomotor dysfunction has not yet been investigated.

**Methods:**

One-hundred and three participants, genotyped for *ALDH2* rs671, were administered a dose of 1.0 g/kg of white spirits. The blood ethanol concentration (BEC) and acetaldehyde concentration (BAAC) were measured at specific time intervals before and after alcohol consumption. Additionally, auditory simple reaction time (ASRT), visual choice reaction time (VCRT), pursuit tracking task (PTT) and digit-symbol substitution test (DSST) were used to evaluate psychomotor function. Linear mixed-effects model was used to analyze the effects of sex and the *ALDH2* genotype on alcohol metabolism and psychomotor function..

**Results:**

Acetaldehyde metabolism depended on both *ALDH2* genotype and sex. Women with ALDH2*1/*1 genotype exhibited 2.21 to 18.27 µmol/L higher BAAC levels than men with the same genotype. Conversely, among participants with ALDH2*1/*2 genotype, BAAC levels of women were 0.25 to 31.32 µmol/L lower than men. The impact of *ALDH2* genotype on psychomotor function varied across the four tests. VCRT increased significantly in men with ALDH2*1/*2 genotype compared to those with ALDH2*1/*1 at 2–4 h post-consumption. In the PTT test, the percentage of time on target decreased by 3.83% and 3.11% in women relative to men at 1 and 2 h post-consumption, respectively. Notably, ASRT performance was significantly correlated with BAAC levels. No effects of *ALDH2* genotype and sex were observed on DSST performance.

**Conclusions:**

*ALDH2* genotype and sex independently or interactively contribute to alcohol-related psychomotor impairment.

## Introduction

Alcohol consumption poses a significant threat to global public health [1,2], with youth being particularly vulnerable [3]. The impact is especially pronounced in low- and middle-income countries [4]. While age-standardized alcohol-attributable morbidity rates have recently declined for both sexes, alcohol-attributable mortality has exhibited a consistent downward trend over the past two decades [5]. However, there was a trend toward increased alcohol consumption during the COVID-19 pandemic [6]. Additionally, driving under the influence of alcohol or drugs remains a significant contributor to road traffic crashes worldwide [7]. Alcohol consumption significantly increases the risk of accidents, including falls, drownings and other unintended incidents [8]. It can also contribute to violent altercations or conflicts [9]. These effects are primarily due to alcohol’s bioactive properties, which impair cognitive function [10], reduce inhibitory control [11] and compromise decision-making accuracy [12].

Individuals exhibit different responses following alcohol consumption, a phenomenon that can be attributed to multiple factors. These factors encompass many individual characteristics, such as alcohol metabolizing enzymes, sex, physical condition and the history of alcohol consumption and food intake during alcohol consumption [13]. These individual differences may give rise to disparities in alcohol metabolism and the subsequent psychophysiological responses of the body to alcohol and its principal metabolites.

Alcohol-related accidents are more common among men than women, according to a road accident survey. Friedman et al. reported that alcohol consumption impairs perceptual judgment in men and women, with men exhibiting a higher increase in errors than women [14]. However, women are more impaired than men in various psychomotor tests, including visual stimuli processing [15], divided attention [16] and pursuit tracking tasks [17], after consuming equal amounts of alcohol. Previous studies investigating gender differences in alcohol-induced performance reported inconsistent findings.

The activity of alcohol-metabolizing enzymes substantially influences the variability of impairment. Men metabolize alcohol more efficiently than women because they have alcohol dehydrogenase (ADH) in the stomach and highly active ADH in the liver, which results in increased blood ethanol concentration (BEC) in women than in men after consuming an equal amount of alcohol [18]. BEC correlates with the severity of the impairment. Aldehyde dehydrogenase 2 (ALDH2) is an additional essential enzyme for metabolizing alcohol. A deficiency in the enzymatic activity of ALDH2, which is formed with ALDH2 subunits encoded by the mutant ALDH2*2 allele (rs671 G > A), leads to the accumulation of acetaldehyde [19]. Our prior research, along with a study by Korean scholars [20,21], demonstrates that individuals carrying the ALDH2*2 mutation experience impaired psychomotor function following alcohol consumption.

Sex and the *ALDH2* genotype are associated with alcohol metabolism and post-alcoholic psychomotor function impairment. Hence, we pose several questions: Is there a combined effect between sex and the *ALDH2* genotype? Will women with ALDH2 deficiency exhibit more severe impairment and an increased propensity for traffic accidents following alcohol consumption?

Binge drinking is a prevalent pattern of excessive alcohol consumption, particularly among young adults and college students. It is defined as consuming a large quantity of alcohol in a short period and has been found to significantly impair psychomotor function [22]. This study investigates the relationship between sex and the *ALDH2* genotype while controlling for potential confounding factors, including age and alcohol consumption history. A comprehensive assessment of the interactive effects between sex and the *ALDH2* genotype can be achieved by comparing the changes in alcohol metabolism and psychomotor performance of participants with different sex and *ALDH2* genotypes after binge drinking. The percentage of East Asians (30%–50%) with the ALDH2*2 allele is greater than that of Caucasians (<5%) [23]. Therefore, this study aimed to provide a scientific toxicological explanation for the occurrence of traffic accidents, which can aid in preventing such accidents.

## Materials and methods

### Participants

The participants were recruited through media advertisements and flyers distributed throughout the university. A total of 105 Chinese medical students voluntarily enrolled in the study. The data collection was conducted in phases, with initial participant recruitment starting in January 2022 and concluding in June 2023. During this period, participants were contacted, screened for eligibility, and provided with detailed information about the study procedures. After obtaining consent, testing sessions were scheduled at convenient times for the participants, ensuring comprehensive data acquisition throughout the study duration. Exclusion criteria include (1) participants with the homozygous ALDH2*2/*2 genotype, who cannot tolerate high doses of alcohol; (2) participants who frequently consume alcoholic beverages; (3) participants who experience severe physical discomfort, including loss of consciousness, after consuming an equivalent dose of alcohol; (4) participants who have consumed alcohol, illicit drugs or medications within one month before the study.

All participants were evaluated using clinical interviews, physical and laboratory examinations and psychiatric assessments with a Chinese version of the Minnesota Multiphasic Personality Inventory-2, the Alcohol Use Disorders Identification Test (AUDIT), and the Michigan Alcoholism Screening Test (MAST). Their urine samples were tested for the presence of nine commonly abused drugs using the Triage^®^ drugs of abuse panel (Alere Inc., USA).

Participants with health issues detected during the physical examination, such as abnormal liver or kidney function, were excluded from the study. Additionally, those who scored ≥5 on the AUDIT and ≥3 on the MAST or tested positive on the urine drug screening were excluded.

The Ethics Committee of Sichuan University approved this study (approval number: K2021039). The aim and details of the study were explained to all participants, and they provided written informed consent before participating in the study.

### Procedure

All participants received extensive training on all psychomotor function tests three days before the study. On the day of testing, the participants ate a light breakfast before 8:00 AM and arrived at the testing site at 9:30 AM, where the room was air-conditioned (25 ± 1 °C). All participants were instructed to drink white spirits (Luzhou Laojiao Tequ with 52% alcohol) at a dose of 1.0 g/kg within 30 min. Specifically, before drinking, all participants were informed to maintain a steady drinking pace, aiming to finish at the 30-minute mark, with guidance provided [24]. A research assistant was present during the session to monitor adherence to this guideline. A licensed medical doctor supervised the participants throughout the day of the study.

### Genotyping

The genomic DNA was extracted from blood samples using the Chelex-100 method [25]. The *ALDH2* rs671 SNP polymorphisms were assessed with gDNA using a multiplex SNaPshot method developed in our laboratory [26].

### Alcohol metabolism

#### Determination of ethanol and acetaldehyde in human blood

Heparin-anticoagulated blood sample tubes were used to collect 1 mL of venous blood from each participant before alcohol consumption and at 0.5, 0.75, 1, 1.5, 2, 3, 4, 5, 6, 7 and 8 h after alcohol consumption. We prepared 500 μL of blood using the optimized perchloric acid method; BEC and blood acetaldehyde concentration (BAAC) were evaluated using headspace gas chromatography with flame ionization, as previously described [27]. Specifically, blood samples were treated with 0.5 mol/L perchloric acid, and the supernatants were collected by centrifugation for the subsequent analysis. Ethanol and acetaldehyde concentrations in the blood were then determined using headspace gas chromatography (GC-2010 Plus and HS-20; Shimadzu, Kyoto, Japan), following the methodology outlined by Okada and Mizoi [28].

#### Calculation of ethanol elimination rate

The ethanol elimination rate (β_60_) was calculated using the substitution of BEC between 4 h and 5 h on the descending portion of the BEC curve [29].

### Assessment of psychomotor function

Before and at 1, 2, 3, 4, 5, 6, 7 and 8 h after alcohol consumption, the participants were instructed to perform a series of psychomotor function tests, including auditory simple reaction time (ASRT), visual choice reaction time (VCRT) [30], pursuit tracking task (PTT) [31] and digit-symbol substitution test (DSST). These tests were performed on a computer using the PsyTech platform (Xinyi Electronic Technology Company, Shanghai, China).

#### Auditory simple reaction time

A pure tone at 750 Hz was transmitted to the participants through the speakers for 500 ms. There is a 2-second interval between the two tones. They were instructed to press the button immediately after the stimulus was presented. The mean reaction durations for 10 stimulus presentations were recorded.

#### Visual choice reaction time

Participants were shown one of the three circles that appeared randomly on the screen in red, green and yellow colours. Participants were directed to press buttons corresponding to three different colours. A 1-second interval separates the two stimulus presentations. The mean reaction durations for 20 stimulus presentations were recorded.

#### Pursuit tracking task

Participants were shown a red circle with a diameter of 1.5 cm on the screen. They were instructed to keep their mouse cursor on the red circle as it moved steadily along a circular path for as long as possible during a 30-s trial. The movement speed of red circle is 15 laps per minute. The duration the cursor remained on target was recorded.

#### Digit-symbol substitution test

Participants were given a key grid comprising corresponding symbols and numbers and a test section containing empty boxes and numbers. They were instructed to fill as many empty boxes as possible with a symbol that matched each number. The number of correct substitutions achieved within 90 s was recorded.

### Statistical analysis

The linear mixed-effects models were used to analyze the effects of sex and the *ALDH2* genotype on alcohol metabolism and psychomotor function. Six separate models were calculated using the following parameters: BEC, BAAC, ASRT, VCRT, PTT and DSST. For BEC and BAAC, repeated measures were performed at 0.5, 0.75, 1, 1.5, 2, 3, 4, 5, 6, 7 and 8 h after alcohol consumption. For ASRT, VCRT, PTT and DSST, repeated measures were performed at 1, 2, 3, 4, 5, 6, 7 and 8 h. In addition to sex (women vs. men), *ALDH2* genotype (ALDH2*1/*1 vs. ALDH2*1/*2), and the measurement time after last alcohol consumption, each model incorporated the interaction between these three variables. To account for potential influences on subsequent measurements, baseline values of ASRT, VCRT, PTT and DSST before alcohol consumption were incorporated as covariates in the respective analyses. All biologically plausible interactions, including sex × time, *ALDH2* × time, *ALDH2* × sex, and sex × *ALDH2* × time, were tested. If there was no significant interaction, a reduced model was run. The linear mixed-effects model was constructed for each outcome to estimate the size of interactive effects or main effects, fitted with restricted maximum likelihood estimation and an unstructured covariance matrix. In the post-hoc comparison, least-squares adjusted means were calculated for all four combinations grouped by sex and *ALDH2* type at each time point. If the interaction of sex, genotype and time was significant, between-group differences (Women vs. Men or ALDH2*1*1 vs. ALDH2*1/*2) and its corresponding 95% confidence intervals at each time point were estimated. If there was no interaction related to sex or genotype, the main effect of sex or genotype was estimated. The *P*-value was adjusted using a Bonferroni correction to account for multiple model comparisons where necessary. Sensitivity analyses were conducted to control for potential confounding effects of age and alcohol consumption history, and to assess the robustness of the main findings.

A multivariate linear regression model with sex and *ALDH*2 genotype was constructed for the alcohol elimination rate. Pearson’s correlation coefficients were calculated between each pair of Z-score transformed BAAC/BEC and psychomotor function performance, measured at 1 and 2 h (the time point with the highest BAAC and BEC, or poorest psychomotor function) to determine the associations between psychomotor performance and blood concentrations of ethanol and acetaldehyde.

R software (v4.0.1), with the main packages ‘lmerTest’ and ‘emmeans’, were used for all analyses. *p* < 0.05 was considered statistically significant. Additionally, post-hoc power analyses were conducted for each study outcome using GLIMMPSE tools (v3.1.3).

## Results

**Figure 1.**
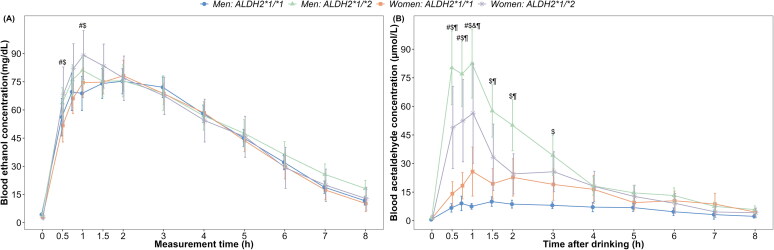
Means and 95% confidence intervals of BAC and BAAC grouped by sex and *ALDH2* type at each time point. The values of alcohol metabolism parameter by each group are shown for (A) blood ethanol concentration (BEC) and (B) blood acetaldehyde concentration (BAAC). All *p*-values reflect Bonferroni corrections. The symbol # indicates *p* < 0.05 for a significant difference between ALDH2*1*1 and ALDH2*1*2 groups among women participants at the time point. The symbol $ indicates *p* < 0.05 for a significant difference between ALDH2*1*1 and ALDH2*1*2 groups among men at the time point. The symbol & indicates *p* < 0.05 for a significant difference between women and men among participants with ALDH2*1*1 type. The symbol ¶ indicates *p* < 0.05 for a significant difference between women and men among participants with ALDH2*1*2 type.

**Figure 2.**
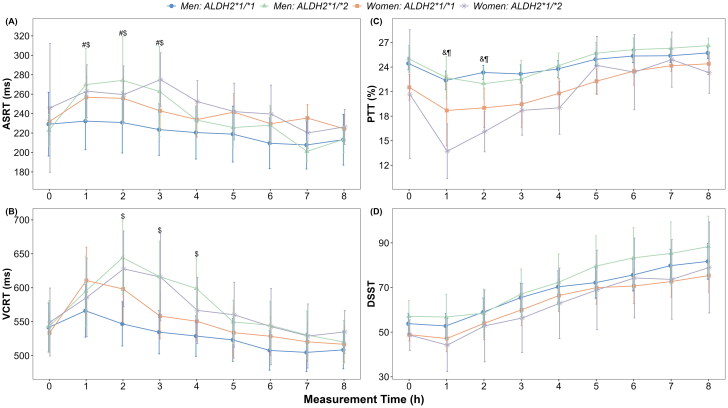
Means and 95% confidence intervals of psychomotor function parameters grouped by sex and *ALDH2* type at each time point. The values of psychomotor function parameters by each group are shown for (A) auditory simple reaction time (ASRT), (B) visual choice reaction time (VCRT), (C) pursuit tracking task (PTT) and (D) digit symbol substitution test (DSST). All comparisons are controlled for baseline values. All *p*-values reflect Bonferroni corrections. The symbol # indicates *p* < 0.05 for a significant difference between ALDH2*1*1 and ALDH2*1*2 groups among women participants at the time point. The symbol $ indicates *p* < 0.05 for a significant difference between ALDH2*1*1 and ALDH2*1*2 groups among men at the time point. The symbol & indicates *p* < 0.05 for a significant difference between women and men among participants with ALDH2*1*1 type. The symbol ¶ indicates *p* < 0.05 for a significant difference between women and men among participants with ALDH2*1*2 type.

A total of 67 participants with the ALDH2*1/*1 genotype and 43 participants with the ALDH2*1/*2 genotype were enrolled in this study. Two individuals with an Alcohol Use Disorders Identification Test (AUDIT) score of ≥5 were excluded. Additionally, during the alcohol consumption experiment, four women and one man with the ALDH2*1/*2 genotype experienced vomiting one or more times after drinking and were also excluded from the statistical analysis. Consequently, data from 103 participants were included in the final analysis, comprising 43 women (ALDH2*1/*1: n = 30, ALDH2*1/*2: n = 13) and 60 men (ALDH2*1/*1: n = 35, ALDH2*1/*2: n = 25). Table 1 shows the participant characteristics, categorized by sex and *ALDH2* genotype. No significant differences were observed among the four groups according to age, body mass index (BMI), AUDIT scores and MAST scores. Apart from PTT levels, no significant differences were observed between groups at baseline according to ASRT, VCRT or DSST (Table 1). The final model for each study outcome was displayed in Tables S1 and S2. Post-hoc power analyses demonstrated that we achieved over 80% power at a significance level of 0.05 to detect the effects of sex and genotype on alcohol metabolism and psychomotor performance. We analyzed the interactive effects or main effects and compared the effect estimates at each time point between groups, especially on the sex or *ALDH2* genotype. Figure 1 and Figure 2 showed the observed means of all study outcomes for four groups. Supplementary Figure 1 showed the individual values of each study outcome. Supplementary Figures 2–7 and Tables S3–S8 were the results of estimated interactive effects across time or main effects of sex and genotype.

**Table 1.** Subject characteristics, mean ± SD.

Characteristics	Women		Men	
	ALDH2*1/*1 (*n* = 30)	ALDH2*1/*2 (*n* = 13)	ALDH2*1/*1 (*n* = 35)	ALDH2*1/*2 (*n* = 25)
Age (y)	22.50 ± 1.59	23.15 ± 2.80	22.03 ± 2.57	22.96 ± 1.95
BMI (kg/m^2^)	20.08 ± 1.68	21.53 ± 4.53	22.24 ± 3.10	21.98 ± 2.93
AUDIT (scores)	1.87 ± 1.67	1.31 ± 0.72	2.03 ± 1.72	1.52 ± 1.10
MAST (scores)	0.97 ± 1.45	0.54 ± 1.05	1.28 ± 1.09	0.79 ± 1.49
ASRT	231.90 ± 41.38	245.92 ± 121.97	208.92 ± 36.83	222.86 ± 41.25
VCRT	533.45 ± 81.83	536.87 ± 91.60	541.55 ± 108.95	543.13 ± 97.40
PTT^a^	21.03 ± 3.02	20.29 ± 5.08	24.45 ± 2.14	25.01 ± 3.25
DSST	49.46 ± 7.99	48.80 ± 12.25	54.48 ± 13.35	57.00 ± 16.69

^a^ The differences were observed between groups at baseline.

Abbreviations: ASRT = auditory simple reaction time, unit = ms; AUDIT = the Alcohol Use Disorders Identification Test; BMI = body mass index; DSST = digit symbol substitution test, unit = number of correct match; MAST = the Michigan Alcoholism Screening Test; PTT = pursuit tracking task, unit = percentage of time on the target(%); SD = standard deviation; VCRT = visual choice reaction time, unit = ms.

**Table 2.** Results from multivariate linear regression model for alcohol disappearance rate (β_60_).

Variables	Estimate	95% CI	*P*
Intercept	13.34	0.69	<0.001
Sex (ref: women)			
Men	−1.39	−3.06 to 0.28	0.107
*ALDH2* type (ref: ALDH2*1/*1)			
ALDH2*1/*2	−1.96	−3.67 to −0.25	0.026

Abbreviation: CI = confidence interval.

**Table 3.** Pearson correlation coefficients (95%CI) between psychomotor function and blood ethanol concentration and blood acetaldehyde concentration levels at 1 h and 2 h.

	ASRT	VCRT	PTT	DSST
1h BEC	0.163 (–0.033 to 0.347)	0.145 (−0.053 to 0.332)	−0.176 (−0.433 to 0.107)	−0.066 (−0.270 to 0.144)
2h BEC	0.033 (−0.168 to 0.231)	−0.069 (−0.266 to 0.134)	−0.218 (−0.468 to 0.064)	−0.177 (−0.374 to 0.037)
1h BAAC	0.238 (0.042 to 0.416)*	0.104 (−0.097 to 0.296)	−0.120 (−0.390 to 0.170)	0.001 (−0.209 to 0.212)
2h BAAC	0.124 (−0.077 to 0.316)	0.168 (−0.034 to 0.356)	−0.185 (−0.441 to 0.098)	0.064 (−0.150 to 0.272)

Abbreviations: ASRT = auditory simple reaction time; BAAC = blood acetaldehyde concentration; BEC = blood ethanol concentration; CI = confidence interval; DSST = digit symbol substitution test; PTT = pursuit tracking task; VCRT = visual choice reaction time.

* *p* < 0.05.

### Effects of sex and ALDH2 genotype on alcohol metabolism

The results revealed no apparent sex × *ALDH2* × time interaction, sex × *ALDH2* interaction, sex × time interaction and main effect of sex for BEC (Table S1). However, a significant two-way interaction effect was observed between *ALDH2* genotype and time on ethanol metabolism (F_(10, 980.43)_ = 3.41, *p* < 0.001, Table S1), indicating that the relationship between *ALDH2* genotype and BEC varied over time after alcohol consumption. Specifically, BEC was significantly higher in the ALDH2*1/*2 group than in the ALDH2*1/*1 group at 0.5 and 1 h after alcohol consumption (all *P*-_Bonferroni_<0.05; Figure 1A and Supplementary Figure 2). No differences were observed in BEC between women and men. A significant three-way interaction effect was observed among sex, *ALDH2* genotype and time (F_(10, 988.06)_ = 5.71, *p* < 0.001, Table S1). The results of between-group difference comparisons were shown in Figure 1B and Supplementary Figure 3. In the ALDH2*1/*2 group, women had significantly lower BAAC than men, with statistical significance observed from 0.5 h to 2 h after alcohol consumption (all *P*-_Bonferroni_<0.05). Conversely, in the ALDH2*1/*1 group, women had higher BAAC than men, and this difference was significant at 1 h with a Bonferroni-correction *p* < 0.05. Among the women, BAAC levels were significantly higher in the ALDH2*1/*2 group than in the ALDH2*1/*1 group at 0.5, 0.75, 1 and 1.5 h (all *P*-_Bonferroni_<0.05). Among the men, BAAC levels were significantly higher in the ALDH2*1/*2 group than in the ALDH2*1/*1 group from 0.5 to 3 h after alcohol consumption (all *P*-_Bonferroni_<0.05). The results for BEC and BAAC levels remained consistent with the primary analyses when adding age and alcohol consumption history as covariates in the corresponding models (see in Supplementary Tables S9 and S10).

The β_60_ was influenced by the *ALDH2* genotype. The average β_60_ of participants with the ALDH2*1/*1 genotype was 1.96 times higher than that of those with the ALDH2*1/*2 genotype (*p* = 0.026, Table 2). No significant differences were observed in the β_60_ between women and men (*p* = 0.107).

### Assessment of factors influencing psychomotor function

We observed a significant two-way interaction effect of *ALDH2* genotype and time on ASRT (F_(7, 683.80)_ = 4.33, *p* < 0.001, Table S2). There was no evidence for the interaction between sex and *ALDH2* genotype or between sex and time. The ALDH2*1/*2 group exhibited longer reaction times than the ALDH2*1/*1 group, with significant differences observed at 1, 2 and 3 h after alcohol consumption (all *P*-*_Bonferroni_* < 0.05; Figure 2A and Supplementary Figure 4).

The effects of *ALDH2* genotype on VCRT varied across time after alcohol consumption (F_(7, 686.84)_ = 5.61, *p* < 0.001, Table S2) and were also influenced by sex (F_(1, 496.45)_ = 4.44, *p* = 0.038; Table S2). The results of between-group difference comparisons were shown in Figure 2B and Supplementary Figure 5. The estimated VCRT of participants with ALDH2*1/*2 was significantly longer than that of those with ALDH2*1/*1 in the men group at 2, 3 and 4 h after alcohol consumption (*P-_Bonferroni_* < 0.05). However, in women, there was no such difference between ALDH2*1/*2 group and ALDH2*1/*1 group.

The relationship between *ALDH2* genotype and PTT varied across time (F_(7, 492.58)_ = 3.38, *p* = 0.002, Table S2), and the effect of sex on PTT was dependent on time (F_(7, 492.01)_ = 7.31, *p* < 0.001). In both ALDH2*1/*1 group and ALDH2*1/*2 groups, men had a longer time on the target in PTT test than women after alcohol consumption, with significant differences observed at 1 and 2 h (*P-_Bonferroni_* < 0.05; Figure 2C and Supplementary Figure 6). The differences in PTT between ALDH2*1/*1 group and ALDH2*1/*2 group at each time point after alcohol consumption were not statistically significant (all *P-_Bonferroni_* > 0.05).

Additionally, a two-way interaction effect of *ALDH2* genotype and time on DSST was observed (F_(7, 614.21)_ = 3.87, *p* < 0.001, Table S2). The number of correct substitutions was lower in the ALDH2*1/*2 group than in the ALDH2*1/*1 group from 1 to 4 h. However, it was higher in the ALDH2*1/*2 group than in the ALDH2*1/*1 group from 5 to 8 h (Figure 2D and Supplementary Figure 7). The differences in DSST between the ALDH2*1/*2 and ALDH2*1/*1 groups were insignificant at any time point (all *P-_Bonferroni_* > 0.05).

After controlling for age and alcohol consumption history, the results for psychomotor function remained consistent with primary analyses (see in Supplementary Tables S11-S14).

### Correlations between psychomotor function and alcohol metabolism

Results in Table 3 showed the correlations between psychomotor performance and log-transformed BEC and BAAC measured at 30 and 60 min. We found that ASRT was significantly correlated with BAAC at 1 h (*r* = 0.238; 95%CI: 0.042 to 0.416; *p* < 0.005). No significant correlations were found between other psychomotor performances and BAAC or BEC at 1 or 2 h (*p* > 0.005).

## Discussion

Herein, we enrolled 103 participants, including healthy women and men, with different *ALDH2* genotypes. They were young adults with similar ages, BMIs and alcohol consumption patterns. Other factors that could potentially influence the ethanol pharmacokinetics were controlled, including recent alcohol consumption history and gastric filling status. We used repeated measures ANOVA and the linear mixed-effects model to examine the association of gender and *ALDH2* genotype with alcohol metabolism and psychomotor dysfunction. The consistent results from the two methods provided the robustness of our study. Previous studies have shown that alcohol metabolism changes with age, making older adults more sensitive to its toxic effects [32]. Additionally, heavy social drinkers may perceive the impairing effects of alcohol differently than light social drinkers [22,33]. Although individuals who consumed alcohol frequently or within the month prior to the study were excluded, we still conducted sensitivity analyses to control for potential confounding effects of age and alcohol consumption history, and to assess the robustness of our main findings.

### Alcohol metabolism

In this experiment, participants consumed peanuts and other snacks concurrently with alcohol intake, resulting in a delayed transit of alcohol to the small intestine and subsequent absorption into the bloodstream. Consequently, the BEC did not increase sharply. We found that the mean BEC level in participants with ALDH2*1/*2 was significantly higher than those with ALDH2*1/*1 from 0.5 to 1 h after alcohol administration. Participants with the ALDH2*2 allele developed a deficiency in ALDH2 enzyme activity, which resulted in the accumulation of acetaldehyde in the blood after alcohol consumption. This metabolic impairment indirectly affects the disappearance rate of ethanol, consequently giving rise to BEC. Participants with ALDH2*1/*1 had a higher β_60_ than those with ALDH2*1/*2.

Nevertheless, no statistically significant differences in BEC were observed between sexes following alcohol consumption in the present study. However, previous studies reported that women attain higher BEC levels than men after consuming equivalent quantities of alcohol [34,35]. The mean value of β_60_ in women was higher than that of men. However, it was not significantly different. In the previous 14 studies that examined sex differences based on β_60_ using moderate alcohol doses (0.3–0.8 g/kg), 10 (including Chinese literature) reported that women reached a higher β_60_ than men, whereas four studies reported no significant sex differences in β_60_ [36,37]. The sex differences of β_60_ disappeared after the rates were corrected for body water, volume of distribution or body weight [38,39]. The doses involved in the studies mentioned above are lower than those in this study. Thus, the lack of sex differences observed can be attributed to the possibility that β_60_ reaches its maximum in both sexes when the dosage is sufficiently high. Previous studies reported that β_60_ was higher in women than in men, suggesting that sex differences in hormonal levels are critical [40]. For instance, elevated serum progesterone levels were associated with faster ethanol elimination rates in women than in men [41].

We observed an interactive effect between *ALDH2* genotype and sex on acetaldehyde metabolism. The BAAC levels in participants with the ALDH2*1/*2 genotype were higher than those in participants with the ALDH2*1/*1 genotype following alcohol consumption. Additionally, divergence in BAAC levels persists in men for up to 3 h after alcohol consumption, whereas in women, this difference subsides rapidly. These findings are consistent with those of similar investigations conducted on Chinese Han and Japanese populations [42].

The sex disparity in various *ALDH2* genotypes yields significant findings. At similar time points, women with the ALDH2*1/*1 genotype had higher blood BAAC levels than men with the same genotype after alcohol consumption. However, women with ALDH2*1/*2 had lower BAAC levels than men with the same genotype. Female rats demonstrated 70% higher hepatic ADH activity than male rats and produced a transient surge of BAAC at levels 2.5 times higher than those observed in male rats [43]. Therefore, the increased ADH enzyme activity in females leads to a rapid conversion of BEC into BAAC after alcohol consumption, but the enzymatic activity of ADH in serum was not detected in the present study. ALDH2 deficiency has a greater impact on BAAC in men with the ALDH2*2 allele than in women with the ALDH2*2 allele. Further research involving ADH enzymatic activity, varying doses and a larger sample size is required to validate our findings.

### Psychomotor dysfunction induced by alcohol

We incorporated a battery of reliable and sensitive tests related to psychomotor performance. These indicators are critical for individuals such as drivers, pilots and security guards. Despite comprehensive training provided to all volunteers regarding all psychomotor function tests, the initial sober performance levels of PTT in men were greater than those in women. Therefore, changes in the psychomotor performance of participants were compared using a linear mixed-effects model after controlling for baseline levels.

The assessment of reaction time is a crucial method for determining the rate at which an individual processes central information and responds to coordinated peripheral movement [44]. We demonstrated that the *ALDH2* genotype affects simple and complex reaction times, which show temporal fluctuations after alcohol consumption. The alterations in both types of ASRT and VCRT follow a U-shape inverted curve, which is consistent with those of previous studies [45]. Individuals with the ALDH2*1/*2 genotype had a longer reaction time than those with the ALDH2*1/*1 genotype. This difference is most significant before and after the peak of BAAC after alcohol consumption. This is when individuals with ALDH2*1/*2 and ALDH2*1/*1 genotypes exhibit the greatest disparity in BAAC. The association between behavioural impairment and BAAC is consistent with those of previous studies conducted on Korean populations [20]. Accordingly, the reaction time is associated with BEC and BAAC. Acetaldehyde can reach the brain through the bloodstream and cross the blood-brain barrier, particularly when BAAC levels are elevated [46]. It acts as a biologically active substance, exhibiting a synergistic interaction with ethanol [47]. Therefore, individuals with ALDH2 deficiency exhibit slower reactions to changes in traffic lights and the sound of horns after consuming alcohol, increasing the likelihood of traffic accidents.

The sex difference was observed in the ASRT test when controlling for the *ALDH2* genotype and time variables. Women exhibited a slower reaction response than men following alcohol consumption. The VCRT test yielded an unexpectedly significant interaction effect between *ALDH2* genotype and sex. Individuals with the ALDH2*1/*2 genotype had a slower reaction response than those with the ALDH2*1/*1 genotype; however, this disparity was observed in men and not in women. Overall, women with ALDH2 deficiency appear to have the longest auditory reaction time among all groups. Additionally, both men and women with the ALDH2*1/*2 genotype have longer reaction times compared to participants with the ALDH2*1/*1 genotype. The auditory stimulus reaches the cortex more rapidly than the visual stimulus [48]. VCRT measures the time it takes to respond to multiple stimuli with different responses, which is more complex than ASRT. Combining these two reasons, VCRT demands higher levels of cognitive processing than ASRT. As discussed earlier, men with the ALDH2*2 variant have temporarily higher BAAC levels than women with the ALDH2*2 variant after alcohol consumption. However, this study did not find a significant correlation between BAAC levels and VCRT impairment.

The pursuit-tracking task is known to be sensitive to motor coordination. Alcohol impairs psychomotor coordination by approximately 25% in PTT studies than in placebo [49]. Herein, a significant reduction in the time spent on the target was observed in the ALDH2*1/*2 group than in the ALDH2*1/*1 group at 2 and 3 h after alcohol consumption. This is also a period when individuals with ALDH2 deficiency have elevated BAAC. Based on gender, women exhibit greater alcohol-induced impairment than men in PTT at some time points, particularly during the earlier stages. Evidence suggests that the effects of alcohol impairment vary depending on the skill level of an individual, with individuals possessing lower skill levels exhibiting more pronounced impairments [49]_._ Therefore, the further study should recruit volunteers with similar proficiency in PTT operation, which can better ensure the feasibility of the results. The digit-symbol substitution test is commonly used to assess general cognitive efficiency, working memory and information processing [50]. During alcohol intoxication, DSST performance is significantly impaired, with scores approximately 10 points lower than those achieved under placebo conditions. No significant differences were observed in DSST performance based on *ALDH2* genotype or sex in this study. Performance improved gradually over time after alcohol consumption. This improvement may be attributed to learning or practice effects, as the same test is administered multiple times, although with random digit-symbol pairings.

This study has several limitations. First, while SNP polymorphisms of *ADH1B* rs1229984, *ADH1C* rs698 and *CYP2E1* rs2031920 were determined simultaneously (data not shown), no significant differences in alcohol metabolism or psychomotor performance were observed due to the limited sample size. Second, the participants were predominantly young, and further confirmation is needed to determine whether the degree of impairment after alcohol consumption is consistent with our findings, as different age groups may experience physiological changes. Third, this study did not assess mood changes after alcohol consumption or examine the relationship between sex, genotype, and mood [51]. Forth, baseline behavioural value discrepancies were observed between the women and men who participated in this study. It is important to conduct preliminary experiments to address gender-related behavioural disparities. In addition, this study exclusively focused on Han Chinese participants, and the tests were conducted among young people, limiting the generalizability of our findings to other populations. Therefore, further investigations are warranted to determine the practical implications, particularly in complex scenarios such as driving behaviours, elderly populations and among alcoholics.

In summary, this study examines the interaction effect of genotype and sex on alcohol metabolism and psychomotor dysfunction resulting from the consumption of a high dose of alcohol. Individuals carrying the ALDH2*1/*2 genotype have been found to exhibit higher levels of BEC compared to those carrying the ALDH2*1/*1 genotype. Additionally, the sex differences in BAAC trends are reversed in individuals with varying *ALDH2* genotypes. Specifically, individuals with the ALDH2*1/*2 genotype experience poorer reaction time and performance on the PTT during specific phases, particularly among men when the VCRT is observed. Furthermore, ASRT exhibits a statistically significant correlation with BAAC. The findings suggest that both sex and genotype play significant roles in alcohol metabolism and psychomotor impairment. Furthermore, their interaction may have additional effects on these outcomes. Individuals with the ALDH2*1/*2 genotype exhibit pronounced impairments in auditory and visual reaction times as well as motor coordination when exposed to a high dose of alcohol. This heightened susceptibility may increase the risk of traffic accidents when compared to individuals with the ALDH2*1/*1 genotype. Notably, after consuming alcohol, men with *ALDH2* deficiency experience more pronounced impairment in visual choice reaction time, while women with limited psychomotor proficiency display notable deficits in motor coordination. Collectively, these factors contribute to an increased propensity for traffic accidents following alcohol consumption. Notably, our study was conducted using a relatively homogeneous sample of Han Chinese individuals. Future investigations should include larger, more diverse populations and scenarios, along with additional assessment tests, to validate and substantiate these observations.

## Supplementary Material

Supplemental Material

## Data Availability

The data will be made available upon reasonable request to the corresponding author.
