# Supplementary material for: Sex- and ALDH2-dependent differences in alcohol metabolism and psychomotor performance: a study in Han Chinese adults after binge drinking
Source: Ann Med. 2025 Apr 28;57(1):2496798. doi: 10.1080/07853890.2025.2496798 (PMC12039403; doi:10.1080/07853890.2025.2496798)
Supplement: Supplemental Material [file IANN_A_2496798_SM8626.zip › Sup/Supplementary Figure Legends_25Mar2025.docx]

## Supplementary Figure Legends

**Supplementary Figure 1.** Individual curves of the study outcomes for all the participants. BEC = blood ethanol concentration; BAAC = blood acetaldehyde concentration; ASRT = auditory simple reaction time; VCRT = visual choice reaction time; PTT = pursuit tracking task; DSST = digit symbol substitution test.

**Supplementary Figure 2.** The estimated differences in least-square adjusted means of BEC between groups at each time point. BEC, blood ethanol concentration; LSMs, least-square means. Note: There was no sex*time interaction or sex**ALDH2* type interaction.

**Supplementary Figure 3.** The estimated differences in least-square adjusted means of BAAC between groups at each time point. BAAC, blood acetaldehyde concentration; LSMs, least-square means.

**Supplementary Figure 4.** The estimated differences in least-square adjusted means of ASRT between groups at each time point. ASRT, auditory simple reaction time; LSMs, least-square means. Note: There was no sex*time interaction or sex**ALDH2* type interaction.

**Supplementary Figure 5.** The estimated differences in least-square adjusted means of VCRT between groups at each time point. VCRT, visual choice reaction time; LSMs, least-square means. Note: There was no sex*time interaction.

**Supplementary Figure 6.** The estimated differences in least-square adjusted means of PTT between groups at each time point. PTT, pursuit tracking task; LSMs, least-square means. Note: There was no sex**ALDH2* type interaction.

**Supplementary Figure 7.** The estimated differences in least-square adjusted means of DSST between groups stratified by sex and ALDH2 type at each time point. DSST, digit symbol substitution test; LSMs, least-square means. Note: There was no sex*time interaction or sex**ALDH2* type interaction.
