# Supplementary material for: Sex- and ALDH2-dependent differences in alcohol metabolism and psychomotor performance: a study in Han Chinese adults after binge drinking
Source: Ann Med. 2025 Apr 28;57(1):2496798. doi: 10.1080/07853890.2025.2496798 (PMC12039403; doi:10.1080/07853890.2025.2496798)
Supplement: Supplemental Material [file IANN_A_2496798_SM8626.zip › Sup/Supplementary Tables_25Mar2025.docx]

## Supplementary Tables

For both BEC and BAAC as shown in Table S1, *Time* is usually the most common and often the strongest factor, indicating that the measurement time points are crucial for changes in blood ethanol and acetaldehyde levels. *Sex* and *ALDH2* genotype also significantly affect blood ethanol or acetaldehyde levels to varying degrees. Certain interactions (especially *Time × ALDH2*) are significant, suggesting that genotype interacts with time to determine ethanol or acetaldehyde metabolism. Only those factors or interactions with *p* < 0.05 (or < 0.001) are considered statistically significant; factors or interactions with *p* > 0.05 are not significant. Post-hoc Power provides an estimate of the test’s power given the model and sample size, indicating the likelihood of detecting significant effects if they truly exist.

## Table S1 Results from type III analysis of variance table with Satterthwaite's method for ethanol.

| Outcomes | Source | Sum of Squares | Mean Squares | NumDF | DenDF | F | *P* | Post-hoc Power |
| --- | --- | --- | --- | --- | --- | --- | --- | --- |
| BEC | Time | 503118.94 | 50311.89 | 10 | 980.43 | 301.73 | **<0.001** | 100% |
|  | Sex | 3.86 | 3.86 | 1 | 99.25 | 0.02 | 0.879 | 5% |
|  | *ALDH2* | 330.58 | 330.58 | 1 | 99.11 | 1.98 | 0.162 | 5% |
|  | Time × *ALDH2* | 5690.8 | 569.08 | 10 | 980.43 | 3.41 | **<0.001** | 96% |
| BAAC | Time | 176165.5 | 17616.55 | 10 | 988.06 | 72.8 | **<0.001** | 100% |
|  | *ALDH2* | 11758.71 | 11758.71 | 1 | 99.01 | 48.6 | **<0.001** | 100% |
|  | Sex | 160 | 160 | 1 | 99.01 | 0.66 | 0.418 | 33.8% |
|  | Time × *ALDH2* | 104845.41 | 10484.54 | 10 | 988.06 | 43.33 | **<0.001** | 100% |
|  | Time × Sex | 5785.17 | 578.52 | 10 | 988.06 | 2.39 | 0.008 | 61.7% |
|  | Sex × *ALDH2* | 2978.19 | 2978.19 | 1 | 99.01 | 12.31 | **0.001** | 100% |
|  | Time × Sex × *ALDH2* | 13813.53 | 1381.35 | 10 | 988.06 | 5.71 | **<0.001** | 100% |

BEC: blood ethanol concentration; BAAC: blood acetaldehyde concentration; NumDF=the number of degrees of freedom in the model; DenDF=the number of degrees of freedom associated with the model errors.

For psychomotor tests as shown in Table S2, *Time* is a significant factor for all four tests (ASRT, VCRT, PTT, DSST), meaning that, as time progresses, there are systematic changes in psychomotor function test performance. *Sex* shows a significant effect only in VCRT (visual choice reaction time) and is not significant in the other three tests (ASRT, PTT, DSST). *ALDH2* genotype has a significant main effect only in DSST and shows a significant interaction with time in DSST, but is not significant for ASRT, VCRT, or PTT. Triple interaction (*Time × Sex × ALDH2*) is not significant for any of the tests, indicating that there is no notable three-way effect across these factors. In other words, *Time* is the primary influencing factor for all psychomotor function tests; *Sex* mainly matters for VCRT; and *ALDH2* genotype primarily affects DSST and interacts with *Time* in determining the pattern of DSST performance changes over time.

## Table S2 Results from type III analysis of variance table with Satterthwaite's method for psychomotor function tests.

| Outcomes | Source | Sum of Squares | Mean Squares | NumDF | DenDF | F | *P* | Post-hoc Power |  |
| --- | --- | --- | --- | --- | --- | --- | --- | --- | --- |
| ASRT | Time | 174486.91 | 24926.70 | 7 | 683.79 | 18.24 | **<0.001** | 100% |  |
|  | *ALDH2* | 9840.17 | 9840.17 | 1 | 97.43 | 7.20 | **0.009** | 100% |  |
|  | Sex | 9558.96 | 9558.96 | 1 | 97.37 | 7.00 | **0.010** | 100% |  |
|  | Baseline ASRT | 27436.06 | 27436.06 | 1 | 97.19 | 20.08 | **<0.001** | 100% |  |
|  | Time × *ALDH2* | 41375.44 | 5910.78 | 7 | 683.80 | 4.33 | **<0.001** | 95.2% |  |
| VCRT | Time | 704799.94 | 100685.71 | 7 | 686.84 | 38.14 | **<0.001** | 100% |  |
|  | *ALDH2* | 14206.59 | 14206.59 | 1 | 96.47 | 5.38 | **0.022** | 96.7% |  |
|  | Sex | 1103.57 | 1103.57 | 1 | 96.43 | 0.42 | 0.519 | 8.16% |  |
|  | Baseline VCRT | 446795.28 | 446795.28 | 1 | 96.04 | 169.27 | **<0.001** | 100% |  |
|  | Time × *ALDH2* | 103594.77 | 14799.25 | 7 | 686.84 | 5.61 | **<0.001** | 92.8% |  |
|  | *ALDH2* × Sex | 11718.35 | 11718.35 | 1 | 496.45 | 4.44 | **0.038** | 86.3% |  |
| PTT | Time | 1887.13 | 269.59 | 7 | 492.59 | 75.32 | **<0.001** | 100% |  |
|  | *ALDH2* | 2.21 | 2.21 | 1 | 71.03 | 0.62 | 0.434 | 56.5% |  |
|  | Sex | 33.90 | 33.90 | 1 | 70.29 | 9.47 | **0.003** | 100% |  |
|  | Baseline PTT | 228.18 | 228.18 | 1 | 69.97 | 63.75 | **<0.001** | 100% |  |
|  | Time × *ALDH2* | 82.13 | 11.73 | 7 | 492.58 | 3.28 | **0.002** | 83.6 |  |
|  | Time × Sex | 183.12 | 26.16 | 7 | 492.01 | 7.31 | **<0.001** | 99.9% |  |
| DSST | Time | 68494.43 | 9784.92 | 7 | 614.20 | 186.69 | **<0.001** | 100% |  |
|  | *ALDH2* | 0.75 | 0.75 | 1 | 88.09 | 0.01 | 0.905 | 23.5% |  |
|  | Sex | 14.45 | 14.45 | 1 | 88.05 | 0.28 | 0.601 | 75.4% |  |
|  | Baseline DSST | 4548.42 | 4548.42 | 1 | 87.99 | 86.78 | **<0.001** | 100% |  |
|  | Time × *ALDH2* | 1421.08 | 203.01 | 7 | 614.21 | 3.87 | **<0.001** | 91.6% |  |

ASRT = auditory simple reaction time; VCRT = visual choice reaction time; PTT = pursuit tracking task; DSST = digit symbol substitution test. *NumDF=the number of degrees of freedom in the model; ^#^DenDF=the number of degrees of freedom associated with the model errors.

Table S3-S8 showed the coefficients of main effects (e.g., Time = 0.5h, *ALDH2*1/*2 vs. ALDH2*1/*1*, Male vs. Female, etc) and interactive effects (e.g., Time=0.75h(*ALDH2*1/*2* vs *ALDH2*1/*1*)). These coefficients indicate the difference relative to the reference level when all other factors remain at their reference levels. For example, the Estimate for “*ALDH2*1/*2* vs. *ALDH2*1/*1*” in Table S3 is positive and significant; thus it indicates that, at the same time and sex (i.e., holding other variables constant), individuals with *ALDH2*1/*2* have a higher average BEC than those with ALDH21/*1. In addition, “Time = 1h × (*ALDH2*1/*2 vs. ALDH2*1/*1)”* indicates the additional effect of having genotype *ALDH2*1/*2* at 1 hour, compared to the reference genotype, beyond what is already captured by the main effects. Interaction coefficients should not be interpreted in isolation; they must be considered in conjunction with the main effects. They show whether there is an additional (incremental or decremental) effect under specific combinations of time, genotype, or sex. Therefore, we have used estimated marginal means of the outcomes when comparing the differences among participants with different sex or genotype.

## Table S3 Results of BEC from multivariate linear mixed-effects model.

| **Variables** | ***β*** | **SE** | **DF** | ***t*** | ***P*** |
| --- | --- | --- | --- | --- | --- |
| Intercept | 53.77 | 2.89 | 192.98 | 18.59 | <0.001 |
| Time=0.75h | 14.05 | 2.31 | 980.84 | 6.07 | <0.001 |
| Time=1h | 17.45 | 2.28 | 980.46 | 7.64 | <0.001 |
| Time=1.5h | 20.35 | 2.3 | 980.72 | 8.83 | <0.001 |
| Time=2h | 22.45 | 2.31 | 980.88 | 9.7 | <0.001 |
| Time=3h | 16.46 | 2.28 | 980.46 | 7.21 | <0.001 |
| Time=4h | 4.24 | 2.29 | 980.64 | 1.85 | 0.065 |
| Time=5h | -9.75 | 2.29 | 980.58 | -4.25 | <0.001 |
| Time=6h | -22.92 | 2.29 | 980.64 | -9.99 | <0.001 |
| Time=7h | -35.84 | 2.28 | 980.46 | -15.69 | <0.001 |
| Time=8h | -42.92 | 2.31 | 980.63 | -18.56 | <0.001 |
| *ALDH2*1/*2* vs *ALDH2*1/*1* | 10.99 | 4.02 | 279.31 | 2.73 | 0.007 |
| Male vs Female | 0.46 | 2.99 | 99.25 | 0.15 | 0.879 |
| Time=0.75h(*ALDH2*1/*2* vs *ALDH2*1/*1*) | -0.97 | 3.85 | 980.82 | -0.25 | 0.802 |
| Time=1h(*ALDH2*1/*2* vs *ALDH2*1/*1*) | 0.64 | 3.83 | 980.7 | 0.17 | 0.867 |
| Time=1.5h(*ALDH2*1*/*2 vs *ALDH2*1/*1*) | -7.5 | 3.82 | 980.65 | -1.96 | 0.05 |
| Time=2h(*ALDH2*1/*2* vs *ALDH2*1/*1*) | -11.55 | 3.83 | 980.71 | -3.02 | 0.003 |
| Time=3h(*ALDH2*1/*2* vs *ALDH2*1/*1*) | -13.05 | 3.81 | 980.56 | -3.43 | 0.001 |
| Time=4h(*ALDH2*1/*2* vs *ALDH2*1/*1*) | -13.24 | 3.82 | 980.62 | -3.47 | 0.001 |
| Time=5h(*ALDH2*1/*2* vs *ALDH2*1/*1*) | -8.35 | 3.82 | 980.6 | -2.19 | 0.029 |
| Time=6h(*ALDH2*1/*2* vs *ALDH2*1/*1*) | -8.23 | 3.82 | 980.62 | -2.16 | 0.031 |
| Time=7h(*ALDH2*1/*2* vs *ALDH2*1/*1*) | -5.42 | 3.81 | 980.56 | -1.42 | 0.155 |
| Time=8h(*ALDH2*1/*2* vs *ALDH2*1/*1*) | -5.75 | 3.83 | 980.61 | -1.5 | 0.134 |

BEC: blood ethanol concentration.

## Table S4 Results of BAAC from multivariate linear mixed-effects model.

| **Variables** | ***β*** | **SE** | **DF** | ***t*** | ***P*** |
| --- | --- | --- | --- | --- | --- |
| Intercept | 14.17 | 3.86 | 351.42 | 3.67 | <0.001 |
| Time=0.75h | 4.19 | 4.02 | 988.03 | 1.04 | 0.297 |
| Time=1h | 11.62 | 4.02 | 988.03 | 2.89 | 0.004 |
| Time=1.5h | 4.99 | 4.05 | 988.45 | 1.23 | 0.219 |
| Time=2h | 8.32 | 4.05 | 988.45 | 2.05 | 0.04 |
| Time=3h | 4.87 | 4.02 | 988.03 | 1.21 | 0.226 |
| Time=4h | 2.26 | 4.02 | 988.03 | 0.56 | 0.573 |
| Time=5h | -4.67 | 4.02 | 988.03 | -1.16 | 0.245 |
| Time=6h | -3.7 | 4.02 | 988.03 | -0.92 | 0.357 |
| Time=7h | -5.44 | 4.02 | 988.03 | -1.36 | 0.176 |
| Time=8h | -9.67 | 4.02 | 988.03 | -2.41 | 0.016 |
| *ALDH2*1/*2* vs *ALDH2*1/*1* | 34.74 | 7.02 | 351.42 | 4.95 | <0.001 |
| Male vs Female | -7.45 | 5.26 | 351.42 | -1.42 | 0.157 |
| Time=0.75h(*ALDH2*1/*2* vs *ALDH2*1/*1*) | -0.62 | 7.3 | 988.03 | -0.09 | 0.932 |
| Time=1h(*ALDH2*1/*2* vs *ALDH2*1/*1*) | -4.08 | 7.3 | 988.03 | -0.56 | 0.576 |
| Time=1.5h(*ALDH2*1/*2* vs *ALDH2*1/*1*) | -20.65 | 7.33 | 988.16 | -2.82 | 0.005 |
| Time=2h(*ALDH2*1/*2* vs *ALDH2*1/*1*) | -32.66 | 7.33 | 988.16 | -4.46 | <0.001 |
| Time=3h(*ALDH2*1/*2* vs *ALDH2*1/*1*) | -28.08 | 7.3 | 988.03 | -3.84 | <0.001 |
| Time=4h(*ALDH2*1/*2* vs *ALDH2*1/*1*) | -33.2 | 7.3 | 988.03 | -4.55 | <0.001 |
| Time=5h(*ALDH2*1/*2* vs *ALDH2*1/*1*) | -31.56 | 7.3 | 988.03 | -4.32 | <0.001 |
| Time=6h(*ALDH2*1/*2* vs *ALDH2*1/*1*) | -36.11 | 7.3 | 988.03 | -4.94 | <0.001 |
| Time=7h(*ALDH2*1/*2* vs *ALDH2*1/*1*) | -38.88 | 7.3 | 988.03 | -5.32 | <0.001 |
| Time=8h(*ALDH2*1/*2* vs *ALDH2*1/*1*) | -35.09 | 7.3 | 988.03 | -4.8 | <0.001 |
| Time=0.75h(Male vs Female) | -1.84 | 5.47 | 988.03 | -0.34 | 0.737 |
| Time=1h(Male vs Female) | -10.82 | 5.47 | 988.03 | -1.98 | 0.048 |
| Time=1.5h(Male vs Female) | -1.68 | 5.5 | 988.26 | -0.31 | 0.76 |
| Time=2h(Male vs Female) | -6.35 | 5.5 | 988.26 | -1.15 | 0.249 |
| Time=3h(Male vs Female) | -3.5 | 5.47 | 988.03 | -0.64 | 0.523 |
| Time=4h(Male vs Female) | -1.86 | 5.47 | 988.03 | -0.34 | 0.734 |
| Time=5h(Male vs Female) | 4.84 | 5.47 | 988.03 | 0.88 | 0.377 |
| Time=6h(Male vs Female) | 1.68 | 5.47 | 988.03 | 0.31 | 0.759 |
| Time=7h(Male vs Female) | 1.92 | 5.47 | 988.03 | 0.35 | 0.726 |
| Time=8h(Male vs Female) | 5.24 | 5.47 | 988.03 | 0.96 | 0.338 |
| Male: *ALDH2*1/*2* vs *ALDH2*1/*1* | 38.77 | 8.94 | 351.42 | 4.34 | <0.001 |
| Time=0.75h(Male: *ALDH2*1/*2* vs *ALDH2*1/*1*) | -4.95 | 9.3 | 988.03 | -0.53 | 0.595 |
| Time=1h(Male: *ALDH2*1/*2* vs *ALDH2*1/*1*) | 5.52 | 9.3 | 988.03 | 0.59 | 0.553 |
| Time=1.5h(Male: *ALDH2*1/*2* vs *ALDH2*1/*1*) | -5.25 | 9.32 | 988.11 | -0.56 | 0.573 |
| Time=2h(Male: *ALDH2*1/*2* vs *ALDH2*1/*1*) | 0.57 | 9.32 | 988.11 | 0.06 | 0.951 |
| Time=3h(Male: *ALDH2*1/*2* vs *ALDH2*1/*1*) | -19.25 | 9.3 | 988.03 | -2.07 | 0.039 |
| Time=4h(Male: *ALDH2*1/*2* vs *ALDH2*1/*1*) | -29.21 | 9.3 | 988.03 | -3.14 | 0.002 |
| Time=5h(Male: *ALDH2*1/*2* vs *ALDH2*1/*1*) | -34.23 | 9.3 | 988.03 | -3.68 | <0.001 |
| Time=6h(Male: *ALDH2*1/*2* vs *ALDH2*1/*1*) | -28.88 | 9.3 | 988.03 | -3.1 | 0.002 |
| Time=7h(Male: *ALDH2*1/*2* vs *ALDH2*1/*1*) | -30.32 | 9.3 | 988.03 | -3.26 | 0.001 |
| Time=8h(Male: *ALDH2*1/*2* vs *ALDH2*1/*1*) | -34.86 | 9.3 | 988.03 | -3.75 | <0.001 |

BAAC: blood acetaldehyde concentration.

## Table S5 Results of ASRT from multivariate linear mixed-effects model.

| **Variables** | ***β*** | **SE** | **DF** | ***t*** | ***P*** |
| --- | --- | --- | --- | --- | --- |
| Intercept | 185.49 | 16.08 | 113.21 | 11.54 | <0.001 |
| Time=2h | -2.84 | 6.68 | 683.96 | -0.42 | 0.671 |
| Time=3h | -11.14 | 6.59 | 683.23 | -1.69 | 0.091 |
| Time=4h | -17.50 | 6.59 | 683.23 | -2.66 | 0.008 |
| Time=5h | -14.56 | 6.59 | 683.23 | -2.21 | 0.027 |
| Time=6h | -24.66 | 6.59 | 683.23 | -3.74 | <0.001 |
| Time=7h | -23.04 | 6.59 | 683.23 | -3.50 | <0.001 |
| Time=8h | -25.89 | 6.59 | 683.23 | -3.93 | <0.001 |
| *ALDH2*1/*2* vs *ALDH2*1/*1* | 30.17 | 10.19 | 336.04 | 2.96 | 0.003 |
| Men vs Women | -19.00 | 7.18 | 97.37 | -2.65 | 0.010 |
| Baseline ASRT | 0.28 | 0.06 | 97.19 | 4.48 | <0.001 |
| Time=2h(*ALDH2*1/*2* vs *ALDH2*1/*1*) | 4.65 | 10.88 | 683.51 | 0.43 | 0.669 |
| Time=3h(*ALDH2*1/*2* vs *ALDH2*1/*1*) | 11.48 | 10.94 | 683.77 | 1.05 | 0.294 |
| Time=4h(*ALDH2*1/*2* vs *ALDH2*1/*1*) | -9.88 | 10.79 | 683.66 | -0.92 | 0.360 |
| Time=5h(*ALDH2*1/*2* vs *ALDH2*1/*1*) | -21.45 | 10.79 | 683.66 | -1.99 | 0.047 |
| Time=6h(*ALDH2*1/*2* vs *ALDH2*1/*1*) | -10.58 | 10.79 | 683.66 | -0.98 | 0.327 |
| Time=7h(*ALDH2*1/*2* vs *ALDH2*1/*1*) | -36.61 | 10.79 | 683.66 | -3.39 | 0.001 |
| Time=8h(*ALDH2*1/*2* vs *ALDH2*1/*1*) | -23.57 | 10.89 | 684.21 | -2.16 | 0.031 |

ASRT = auditory simple reaction time.

## Table S6 Results of VCRT from multivariate linear mixed-effects model.

| **Variables** | **β** | **SE** | **DF** | **t** | ***P*** |
| --- | --- | --- | --- | --- | --- |
| Intercept | 223.59 | 31.04 | 103.46 | 7.20 | <0.001 |
| Time=2h | -19.82 | 9.18 | 687.10 | -2.16 | 0.031 |
| Time=3h | -42.58 | 9.05 | 686.48 | -4.70 | <0.001 |
| Time=4h | -47.81 | 9.01 | 686.22 | -5.31 | <0.001 |
| Time=5h | -58.62 | 9.01 | 686.22 | -6.50 | <0.001 |
| Time=6h | -69.44 | 9.01 | 686.22 | -7.71 | <0.001 |
| Time=7h | -74.77 | 9.01 | 686.22 | -8.30 | <0.001 |
| Time=8h | -74.45 | 9.01 | 686.22 | -8.26 | <0.001 |
| *ALDH2*1/*2* vs *ALDH2*1/*1* | -22.26 | 20.48 | 167.17 | -1.09 | 0.278 |
| Men vs Women | -30.84 | 12.94 | 96.01 | -2.38 | 0.019 |
| Baseline ASRT | 0.71 | 0.05 | 96.05 | 13.01 | <0.001 |
| Time=2h(*ALDH2*1/*2* vs *ALDH2*1/*1*) | 58.66 | 15.27 | 686.85 | 3.84 | <0.001 |
| Time=3h(*ALDH2*1/*2* vs *ALDH2*1/*1*) | 62.86 | 15.34 | 686.56 | 4.10 | <0.001 |
| Time=4h(*ALDH2*1/*2* vs *ALDH2*1/*1*) | 39.92 | 15.12 | 686.94 | 2.64 | 0.008 |
| Time=5h(*ALDH2*1/*2* vs *ALDH2*1/*1*) | 15.75 | 15.19 | 687.19 | 1.04 | 0.300 |
| Time=6h(*ALDH2*1/*2* vs *ALDH2*1/*1*) | 12.75 | 15.19 | 687.19 | 0.84 | 0.402 |
| Time=7h(*ALDH2*1/*2* vs *ALDH2*1/*1*) | 5.36 | 15.12 | 686.94 | 0.35 | 0.723 |
| Time=8h(*ALDH2*1/*2* vs *ALDH2*1/*1*) | 1.91 | 15.12 | 686.94 | 0.13 | 0.900 |
| Men+*ALDH2*1/*2* vs Women+*ALDH2*1/*1* | 47.20 | 22.40 | 96.45 | 2.11 | 0.038 |

VCRT = visual choice reaction time.

## Table S7 Results of PTT from multivariate linear mixed-effects model.

| **Variables** | ***β*** | **SE** | **DF** | ***t*** | ***P*** |
| --- | --- | --- | --- | --- | --- |
| Intercept | 5.49 | 1.54 | 77.95 | 3.57 | 0.001 |
| Time=2h | 0.94 | 0.54 | 491.88 | 1.75 | 0.081 |
| Time=3h | 2.44 | 0.53 | 491.58 | 4.59 | <0.001 |
| Time=4h | 3.60 | 0.53 | 491.59 | 6.77 | <0.001 |
| Time=5h | 4.67 | 0.53 | 491.81 | 8.79 | <0.001 |
| Time=6h | 5.22 | 0.53 | 491.89 | 9.85 | <0.001 |
| Time=7h | 5.62 | 0.53 | 491.89 | 10.59 | <0.001 |
| Time=8h | 6.74 | 0.53 | 491.89 | 12.71 | <0.001 |
| *ALDH2*1/*2* vs *ALDH2*1/*1* | -1.09 | 0.65 | 256.85 | -1.68 | 0.094 |
| Men vs Women | 3.49 | 0.66 | 192.20 | 5.27 | <0.001 |
| Baseline ASRT | 0.56 | 0.07 | 69.97 | 7.98 | <0.001 |
| Time=2h(*ALDH2*1/*2* vs *ALDH2*1/*1*) | -0.47 | 0.70 | 491.51 | -0.68 | 0.499 |
| Time=3h(*ALDH2*1/*2* vs *ALDH2*1/*1*) | -0.25 | 0.70 | 492.43 | -0.37 | 0.714 |
| Time=4h(*ALDH2*1/*2* vs *ALDH2*1/*1*) | 0.76 | 0.69 | 492.55 | 1.11 | 0.266 |
| Time=5h(*ALDH2*1/*2* vs *ALDH2*1/*1*) | 1.77 | 0.69 | 492.86 | 2.58 | 0.010 |
| Time=6h(*ALDH2*1/*2* vs *ALDH2*1/*1*) | 1.38 | 0.68 | 493.34 | 2.02 | 0.044 |
| Time=7h(*ALDH2*1/*2* vs *ALDH2*1/*1*) | 1.70 | 0.68 | 493.34 | 2.49 | 0.013 |
| Time=8h(*ALDH2*1/*2* vs *ALDH2*1/*1*) | 0.97 | 0.68 | 493.34 | 1.43 | 0.153 |
| Time=2h(Men vs Women) | -0.14 | 0.65 | 491.67 | -0.22 | 0.829 |
| Time=3h(Men vs Women) | -1.87 | 0.64 | 491.70 | -2.91 | 0.004 |
| Time=4h(Men vs Women) | -2.30 | 0.64 | 491.73 | -3.59 | <0.001 |
| Time=5h(Men vs Women) | -2.46 | 0.64 | 492.17 | -3.85 | <0.001 |
| Time=6h(Men vs Women) | -2.43 | 0.64 | 492.33 | -3.80 | <0.001 |
| Time=7h(Men vs Women) | -2.85 | 0.64 | 492.33 | -4.46 | <0.001 |
| Time=8h(Men vs Women) | -3.40 | 0.64 | 492.33 | -5.32 | <0.001 |

PTT = pursuit tracking task.

## Table S8 Results of DSST from multivariate linear mixed-effects model.

| **Variables** | ***β*** | **SE** | **DF** | ***t*** | ***P*** |
| --- | --- | --- | --- | --- | --- |
| Intercept | -12.41 | 7.09 | 90.71 | -1.75 | 0.083 |
| Time=2h | 7.07 | 1.33 | 614.13 | 5.32 | <0.001 |
| Time=3h | 13.15 | 1.32 | 614.04 | 9.99 | <0.001 |
| Time=4h | 18.73 | 1.32 | 614.04 | 14.22 | <0.001 |
| Time=5h | 21.33 | 1.32 | 614.04 | 16.20 | <0.001 |
| Time=6h | 23.41 | 1.32 | 614.04 | 17.78 | <0.001 |
| Time=7h | 26.57 | 1.32 | 614.09 | 20.08 | <0.001 |
| Time=8h | 28.80 | 1.34 | 614.17 | 21.55 | <0.001 |
| *ALDH2*1/*2* vs *ALDH2*1/*1* | -1.18 | 3.86 | 123.94 | -0.31 | 0.760 |
| Men vs Women | -1.83 | 3.49 | 88.05 | -0.53 | 0.601 |
| Baseline ASRT | 1.23 | 0.13 | 87.99 | 9.32 | <0.001 |
| Time=2h(*ALDH2*1/*2* vs *ALDH2*1/*1*) | -1.77 | 2.34 | 614.10 | -0.76 | 0.450 |
| Time=3h(*ALDH2*1/*2* vs *ALDH2*1/*1*) | -2.35 | 2.33 | 614.08 | -1.01 | 0.313 |
| Time=4h(*ALDH2*1/*2* vs *ALDH2*1/*1*) | -0.86 | 2.29 | 614.29 | -0.37 | 0.708 |
| Time=5h(*ALDH2*1/*2* vs *ALDH2*1/*1*) | 3.57 | 2.29 | 614.29 | 1.56 | 0.120 |
| Time=6h(*ALDH2*1/*2* vs *ALDH2*1/*1*) | 5.48 | 2.29 | 614.29 | 2.39 | 0.017 |
| Time=7h(*ALDH2*1/*2* vs *ALDH2*1/*1*) | 3.33 | 2.30 | 614.30 | 1.45 | 0.147 |
| Time=8h(*ALDH2*1/*2* vs *ALDH2*1/*1*) | 5.45 | 2.31 | 614.17 | 2.36 | 0.019 |

DSST = digit symbol substitution test.

**Sensitivity analyses**

To account for baseline confounding factors, including age and alcohol consumption history, we then applied sensitivity analyses with these factors as covariates in the corresponding model for each study outcome. The results were displayed in the Supplementary Tables S9-S14. These results showed that after controlling age and alcohol consumption history, the estimates were still consistent with the main analyses.

**Table S9** The estimated differences in EMMs for BEC between different sex or genotype at each time point post-consumption.

| Comparison | Time (h) | Main analyses | | |  | Analyses with adjustment of  age and alcohol consumption history | | |
| --- | --- | --- | --- | --- | --- | --- | --- | --- |
|  |  | Diff in EMMs | SE | *P** |  | Diff in EMMs | SE | *P** |
| ALDH2*1/*1: Women vs Men | 0.5 | -2.53 | 3.11 | 1 |  | -3.29 | 3.5 | 1 |
| ALDH2*1/*2: Women vs Men | 0.5 | -2.53 | 3.11 | 1 |  | -3.29 | 3.5 | 1 |
| Men: ALDH2*1/*1 vs ALDH2*1/*2 | 0.5 | -9.03 | 4.08 | 0.166 |  | -9.11 | 4.22 | 0.19 |
| Women: ALDH2*1/*1 vs ALDH2*1/*2 | 0.5 | -9.03 | 4.08 | 0.166 |  | -9.11 | 4.22 | 0.19 |
| ALDH2*1/*1: Women vs Men | 0.75 | -2.53 | 3.11 | 1 |  | -3.29 | 3.5 | 1 |
| ALDH2*1/*2: Women vs Men | 0.75 | -2.53 | 3.11 | 1 |  | -3.29 | 3.5 | 1 |
| Men: ALDH2*1/*1 vs ALDH2*1/*2 | 0.75 | -7.5 | 4.07 | 0.398 |  | -7.63 | 4.21 | 0.425 |
| Women: ALDH2*1/*1 vs ALDH2*1/*2 | 0.75 | -7.5 | 4.07 | 0.398 |  | -7.63 | 4.21 | 0.425 |
| ALDH2*1/*1: Women vs Men | 1 | -2.53 | 3.11 | 1 |  | -3.29 | 3.5 | 1 |
| ALDH2*1/*2: Women vs Men | 1 | -2.53 | 3.11 | 1 |  | -3.29 | 3.5 | 1 |
| Men: ALDH2*1/*1 vs ALDH2*1/*2 | 1 | -8.98 | 4.05 | 0.165 |  | -9.16 | 4.19 | 0.179 |
| Women: ALDH2*1/*1 vs ALDH2*1/*2 | 1 | -8.98 | 4.05 | 0.165 |  | -9.16 | 4.19 | 0.179 |
| ALDH2*1/*1: Women vs Men | 1.5 | -2.53 | 3.11 | 1 |  | -3.29 | 3.5 | 1 |
| ALDH2*1/*2: Women vs Men | 1.5 | -2.53 | 3.11 | 1 |  | -3.29 | 3.5 | 1 |
| Men: ALDH2*1/*1 vs ALDH2*1/*2 | 1.5 | -0.96 | 4.05 | 1 |  | -0.99 | 4.18 | 1 |
| Women: ALDH2*1/*1 vs ALDH2*1/*2 | 1.5 | -0.96 | 4.05 | 1 |  | -0.99 | 4.18 | 1 |
| ALDH2*1/*1: Women vs Men | 2 | -2.53 | 3.11 | 1 |  | -3.29 | 3.5 | 1 |
| ALDH2*1/*2: Women vs Men | 2 | -2.53 | 3.11 | 1 |  | -3.29 | 3.5 | 1 |
| Men: ALDH2*1/*1 vs ALDH2*1/*2 | 2 | 3.48 | 4.05 | 1 |  | 3.32 | 4.19 | 1 |
| Women: ALDH2*1/*1 vs ALDH2*1/*2 | 2 | 3.48 | 4.05 | 1 |  | 3.32 | 4.19 | 1 |
| ALDH2*1/*1: Women vs Men | 3 | -2.53 | 3.11 | 1 |  | -3.29 | 3.5 | 1 |
| ALDH2*1/*2: Women vs Men | 3 | -2.53 | 3.11 | 1 |  | -3.29 | 3.5 | 1 |
| Men: ALDH2*1/*1 vs ALDH2*1/*2 | 3 | 5.28 | 4.04 | 1 |  | 4.82 | 4.17 | 1 |
| Women: ALDH2*1/*1 vs ALDH2*1/*2 | 3 | 5.28 | 4.04 | 1 |  | 4.82 | 4.17 | 1 |
| ALDH2*1/*1: Women vs Men | 4 | -2.53 | 3.11 | 1 |  | -3.29 | 3.5 | 1 |
| ALDH2*1/*2: Women vs Men | 4 | -2.53 | 3.11 | 1 |  | -3.29 | 3.5 | 1 |
| Men: ALDH2*1/*1 vs ALDH2*1/*2 | 4 | 5.16 | 4.04 | 1 |  | 4.55 | 4.18 | 1 |
| Women: ALDH2*1/*1 vs ALDH2*1/*2 | 4 | 5.16 | 4.04 | 1 |  | 4.55 | 4.18 | 1 |
| ALDH2*1/*1: Women vs Men | 5 | -2.53 | 3.11 | 1 |  | -3.29 | 3.5 | 1 |
| ALDH2*1/*2: Women vs Men | 5 | -2.53 | 3.11 | 1 |  | -3.29 | 3.5 | 1 |
| Men: ALDH2*1/*1 vs ALDH2*1/*2 | 5 | -0.14 | 4.04 | 1 |  | -0.61 | 4.18 | 1 |
| Women: ALDH2*1/*1 vs ALDH2*1/*2 | 5 | -0.14 | 4.04 | 1 |  | -0.61 | 4.18 | 1 |
| ALDH2*1/*1: Women vs Men | 6 | -2.53 | 3.11 | 1 |  | -3.29 | 3.5 | 1 |
| ALDH2*1/*2: Women vs Men | 6 | -2.53 | 3.11 | 1 |  | -3.29 | 3.5 | 1 |
| Men: ALDH2*1/*1 vs ALDH2*1/*2 | 6 | -0.94 | 4.04 | 1 |  | -1.43 | 4.18 | 1 |
| Women: ALDH2*1/*1 vs ALDH2*1/*2 | 6 | -0.94 | 4.04 | 1 |  | -1.43 | 4.18 | 1 |
| ALDH2*1/*1: Women vs Men | 7 | -2.53 | 3.11 | 1 |  | -3.29 | 3.5 | 1 |
| ALDH2*1/*2: Women vs Men | 7 | -2.53 | 3.11 | 1 |  | -3.29 | 3.5 | 1 |
| Men: ALDH2*1/*1 vs ALDH2*1/*2 | 7 | -4.26 | 4.04 | 1 |  | -4.46 | 4.17 | 1 |
| Women: ALDH2*1/*1 vs ALDH2*1/*2 | 7 | -4.26 | 4.04 | 1 |  | -4.46 | 4.17 | 1 |
| ALDH2*1/*1: Women vs Men | 7.75 | -2.53 | 3.11 | 1 |  | -3.29 | 3.5 | 1 |
| ALDH2*1/*2: Women vs Men | 7.75 | -2.53 | 3.11 | 1 |  | -3.29 | 3.5 | 1 |
| Men: ALDH2*1/*1 vs ALDH2*1/*2 | 7.75 | -4.32 | 4.05 | 1 |  | -4.42 | 4.19 | 1 |
| Women: ALDH2*1/*1 vs ALDH2*1/*2 | 7.75 | -4.32 | 4.05 | 1 |  | -4.42 | 4.19 | 1 |

Note: EMMs = Estimated marginal means; BEC = blood ethanol concentration. * *P* values were corrected for multiple comparison using Bonferroni method.

**Table S10** The estimated differences in EMMs for BAAC between different sex or genotype at each time point post-consumption.

| Comparison | Time (h) | Main analyses | | |  | Analyses with adjustment of  age and alcohol consumption history | | |
| --- | --- | --- | --- | --- | --- | --- | --- | --- |
|  |  | Diff in EMMs | SE | *P* |  | Diff in EMMs | SE | *P* |
| ALDH2*1/*1: Women vs Men | 0.5 | 7.45 | 5.35 | 0.985 |  | 8.35 | 5.78 | 0.896 |
| ALDH2*1/*2: Women vs Men | 0.5 | -31.32 | 7.35 | 0 |  | -29.78 | 7.49 | 0.001 |
| Men: ALDH2*1/*1 vs ALDH2*1/*2 | 0.5 | -73.51 | 5.63 | 0 |  | -73.76 | 5.84 | 0 |
| Women: ALDH2*1/*1 vs ALDH2*1/*2 | 0.5 | -34.74 | 7.13 | 0 |  | -35.63 | 7.24 | 0 |
| ALDH2*1/*1: Women vs Men | 0.75 | 9.29 | 5.35 | 0.498 |  | 9.79 | 5.78 | 0.548 |
| ALDH2*1/*2: Women vs Men | 0.75 | -24.52 | 7.35 | 0.006 |  | -22.99 | 7.49 | 0.014 |
| Men: ALDH2*1/*1 vs ALDH2*1/*2 | 0.75 | -67.94 | 5.63 | 0 |  | -67.99 | 5.84 | 0 |
| Women: ALDH2*1/*1 vs ALDH2*1/*2 | 0.75 | -34.12 | 7.13 | 0 |  | -35.21 | 7.24 | 0 |
| ALDH2*1/*1: Women vs Men | 1 | 18.27 | 5.35 | 0.004 |  | 19.39 | 5.78 | 0.005 |
| ALDH2*1/*2: Women vs Men | 1 | -26.02 | 7.35 | 0.003 |  | -24.48 | 7.49 | 0.007 |
| Men: ALDH2*1/*1 vs ALDH2*1/*2 | 1 | -74.95 | 5.63 | 0 |  | -75.57 | 5.84 | 0 |
| Women: ALDH2*1/*1 vs ALDH2*1/*2 | 1 | -30.66 | 7.13 | 0 |  | -31.69 | 7.24 | 0 |
| ALDH2*1/*1: Women vs Men | 1.5 | 9.13 | 5.37 | 0.542 |  | 10.05 | 5.81 | 0.508 |
| ALDH2*1/*2: Women vs Men | 1.5 | -24.39 | 7.35 | 0.006 |  | -22.85 | 7.49 | 0.015 |
| Men: ALDH2*1/*1 vs ALDH2*1/*2 | 1.5 | -47.61 | 5.63 | 0 |  | -48.07 | 5.84 | 0 |
| Women: ALDH2*1/*1 vs ALDH2*1/*2 | 1.5 | -14.1 | 7.16 | 0.297 |  | -15.17 | 7.26 | 0.224 |
| ALDH2*1/*1: Women vs Men | 2 | 13.79 | 5.37 | 0.064 |  | 14.48 | 5.81 | 0.079 |
| ALDH2*1/*2: Women vs Men | 2 | -25.54 | 7.35 | 0.003 |  | -24 | 7.49 | 0.009 |
| Men: ALDH2*1/*1 vs ALDH2*1/*2 | 2 | -41.42 | 5.63 | 0 |  | -41.93 | 5.84 | 0 |
| Women: ALDH2*1/*1 vs ALDH2*1/*2 | 2 | -2.09 | 7.16 | 1 |  | -3.44 | 7.26 | 1 |
| ALDH2*1/*1: Women vs Men | 3 | 10.95 | 5.35 | 0.247 |  | 12.53 | 5.78 | 0.185 |
| ALDH2*1/*2: Women vs Men | 3 | -8.57 | 7.35 | 1 |  | -7.03 | 7.49 | 1 |
| Men: ALDH2*1/*1 vs ALDH2*1/*2 | 3 | -26.18 | 5.63 | 0 |  | -26.73 | 5.84 | 0 |
| Women: ALDH2*1/*1 vs ALDH2*1/*2 | 3 | -6.66 | 7.13 | 1 |  | -7.17 | 7.24 | 1 |
| ALDH2*1/*1: Women vs Men | 4 | 12.37 | 5.35 | 0.127 |  | 13 | 5.78 | 0.151 |
| ALDH2*1/*2: Women vs Men | 4 | -0.25 | 7.35 | 1 |  | 1.29 | 7.49 | 1 |
| Men: ALDH2*1/*1 vs ALDH2*1/*2 | 4 | -11.1 | 5.63 | 0.295 |  | -11.35 | 5.84 | 0.317 |
| Women: ALDH2*1/*1 vs ALDH2*1/*2 | 4 | 1.52 | 7.13 | 1 |  | 0.36 | 7.24 | 1 |
| ALDH2*1/*1: Women vs Men | 5 | 2.62 | 5.35 | 1 |  | 3.61 | 5.78 | 1 |
| ALDH2*1/*2: Women vs Men | 5 | -1.93 | 7.35 | 1 |  | -0.39 | 7.49 | 1 |
| Men: ALDH2*1/*1 vs ALDH2*1/*2 | 5 | -7.72 | 5.63 | 1 |  | -7.84 | 5.84 | 1 |
| Women: ALDH2*1/*1 vs ALDH2*1/*2 | 5 | -3.18 | 7.13 | 1 |  | -3.84 | 7.24 | 1 |
| ALDH2*1/*1: Women vs Men | 6 | 5.77 | 5.35 | 1 |  | 6.66 | 5.78 | 1 |
| ALDH2*1/*2: Women vs Men | 6 | -4.12 | 7.35 | 1 |  | -2.58 | 7.49 | 1 |
| Men: ALDH2*1/*1 vs ALDH2*1/*2 | 6 | -8.52 | 5.63 | 0.786 |  | -8.79 | 5.84 | 0.799 |
| Women: ALDH2*1/*1 vs ALDH2*1/*2 | 6 | 1.37 | 7.13 | 1 |  | 0.45 | 7.24 | 1 |
| ALDH2*1/*1: Women vs Men | 7 | 5.53 | 5.35 | 1 |  | 6.86 | 5.78 | 1 |
| ALDH2*1/*2: Women vs Men | 7 | -2.92 | 7.35 | 1 |  | -1.38 | 7.49 | 1 |
| Men: ALDH2*1/*1 vs ALDH2*1/*2 | 7 | -4.31 | 5.63 | 1 |  | -4.7 | 5.84 | 1 |
| Women: ALDH2*1/*1 vs ALDH2*1/*2 | 7 | 4.13 | 7.13 | 1 |  | 3.54 | 7.24 | 1 |
| ALDH2*1/*1: Women vs Men | 7.75 | 2.24 | 5.35 | 1 |  | 3.94 | 5.78 | 1 |
| ALDH2*1/*2: Women vs Men | 7.75 | -1.7 | 7.35 | 1 |  | -0.16 | 7.49 | 1 |
| Men: ALDH2*1/*1 vs ALDH2*1/*2 | 7.75 | -3.6 | 5.63 | 1 |  | -4.01 | 5.84 | 1 |
| Women: ALDH2*1/*1 vs ALDH2*1/*2 | 7.75 | 0.35 | 7.13 | 1 |  | 0.09 | 7.24 | 1 |

Note: EMMs = Estimated marginal means; BAAC = blood acetaldehyde concentration. * *P* values were corrected for multiple comparison using Bonferroni method.

**Table S11** The estimated differences in EMMs for ASRT between different sex or genotype at each time point post-consumption.

| Comparison | Time (h) | Main analyses | | |  | Analyses with adjustment of  age and alcohol consumption history | | |
| --- | --- | --- | --- | --- | --- | --- | --- | --- |
|  |  | Diff in EMMs | SE | *P* |  | Diff in EMMs | SE | *P* |
| ALDH2*1/*1: Women vs Men | 1 | 10.97 | 8.43 | 1 |  | 14.75 | 9.47 | 0.736 |
| ALDH2*1/*2: Women vs Men | 1 | 10.97 | 8.43 | 1 |  | 14.75 | 9.47 | 0.736 |
| Men: ALDH2*1/*1 vs ALDH2*1/*2 | 1 | -24.7 | 11.17 | 0.167 |  | -24.39 | 11.49 | 0.209 |
| Women: ALDH2*1/*1 vs ALDH2*1/*2 | 1 | -24.7 | 11.17 | 0.167 |  | -24.39 | 11.49 | 0.209 |
| ALDH2*1/*1: Women vs Men | 2 | 10.97 | 8.43 | 1 |  | 14.75 | 9.47 | 0.736 |
| ALDH2*1/*2: Women vs Men | 2 | 10.97 | 8.43 | 1 |  | 14.75 | 9.47 | 0.736 |
| Men: ALDH2*1/*1 vs ALDH2*1/*2 | 2 | -28.88 | 11.22 | 0.063 |  | -29.62 | 11.55 | 0.065 |
| Women: ALDH2*1/*1 vs ALDH2*1/*2 | 2 | -28.88 | 11.22 | 0.063 |  | -29.62 | 11.55 | 0.065 |
| ALDH2*1/*1: Women vs Men | 3 | 10.97 | 8.43 | 1 |  | 14.75 | 9.47 | 0.736 |
| ALDH2*1/*2: Women vs Men | 3 | 10.97 | 8.43 | 1 |  | 14.75 | 9.47 | 0.736 |
| Men: ALDH2*1/*1 vs ALDH2*1/*2 | 3 | -36.31 | 11.27 | 0.009 |  | -35.26 | 11.6 | 0.016 |
| Women: ALDH2*1/*1 vs ALDH2*1/*2 | 3 | -36.31 | 11.27 | 0.009 |  | -35.26 | 11.6 | 0.016 |
| ALDH2*1/*1: Women vs Men | 4 | 10.97 | 8.43 | 1 |  | 14.75 | 9.47 | 0.736 |
| ALDH2*1/*2: Women vs Men | 4 | 10.97 | 8.43 | 1 |  | 14.75 | 9.47 | 0.736 |
| Men: ALDH2*1/*1 vs ALDH2*1/*2 | 4 | -14.39 | 11.12 | 1 |  | -13.53 | 11.44 | 1 |
| Women: ALDH2*1/*1 vs ALDH2*1/*2 | 4 | -14.39 | 11.12 | 1 |  | -13.53 | 11.44 | 1 |
| ALDH2*1/*1: Women vs Men | 5 | 10.97 | 8.43 | 1 |  | 14.75 | 9.47 | 0.736 |
| ALDH2*1/*2: Women vs Men | 5 | 10.97 | 8.43 | 1 |  | 14.75 | 9.47 | 0.736 |
| Men: ALDH2*1/*1 vs ALDH2*1/*2 | 5 | -3.05 | 11.12 | 1 |  | -1.64 | 11.44 | 1 |
| Women: ALDH2*1/*1 vs ALDH2*1/*2 | 5 | -3.05 | 11.12 | 1 |  | -1.64 | 11.44 | 1 |
| ALDH2*1/*1: Women vs Men | 6 | 10.97 | 8.43 | 1 |  | 14.75 | 9.47 | 0.736 |
| ALDH2*1/*2: Women vs Men | 6 | 10.97 | 8.43 | 1 |  | 14.75 | 9.47 | 0.736 |
| Men: ALDH2*1/*1 vs ALDH2*1/*2 | 6 | -14.42 | 11.12 | 1 |  | -13.64 | 11.44 | 1 |
| Women: ALDH2*1/*1 vs ALDH2*1/*2 | 6 | -14.42 | 11.12 | 1 |  | -13.64 | 11.44 | 1 |
| ALDH2*1/*1: Women vs Men | 7 | 10.97 | 8.43 | 1 |  | 14.75 | 9.47 | 0.736 |
| ALDH2*1/*2: Women vs Men | 7 | 10.97 | 8.43 | 1 |  | 14.75 | 9.47 | 0.736 |
| Men: ALDH2*1/*1 vs ALDH2*1/*2 | 7 | 11.77 | 11.12 | 1 |  | 12.43 | 11.44 | 1 |
| Women: ALDH2*1/*1 vs ALDH2*1/*2 | 7 | 11.77 | 11.12 | 1 |  | 12.43 | 11.44 | 1 |
| ALDH2*1/*1: Women vs Men | 8 | 10.97 | 8.43 | 1 |  | 14.75 | 9.47 | 0.736 |
| ALDH2*1/*2: Women vs Men | 8 | 10.97 | 8.43 | 1 |  | 14.75 | 9.47 | 0.736 |
| Men: ALDH2*1/*1 vs ALDH2*1/*2 | 8 | -0.65 | 11.21 | 1 |  | -0.1 | 11.54 | 1 |
| Women: ALDH2*1/*1 vs ALDH2*1/*2 | 8 | -0.65 | 11.21 | 1 |  | -0.1 | 11.54 | 1 |

Note: EMMs = Estimated marginal means; ASRT = auditory simple reaction time, ms. * *P* values were corrected for multiple comparison using Bonferroni method.

**Table S12** The estimated differences in EMMs for VCRT between different sex or genotype at each time point post-consumption.

| Comparison | Time (h) | Main analyses | | |  | Analyses with adjustment of  age and alcohol consumption history | | |
| --- | --- | --- | --- | --- | --- | --- | --- | --- |
|  |  | Diff in EMMs | SE | *P* |  | Diff in EMMs | SE | *P* |
| ALDH2*1/*1: Women vs Men | 1 | 30.96 | 13.2 | 0.126 |  | 28.56 | 15.17 | 0.377 |
| ALDH2*1/*2: Women vs Men | 1 | -11.66 | 18.62 | 1 |  | -13.42 | 19.54 | 1 |
| Men: ALDH2*1/*1 vs ALDH2*1/*2 | 1 | -22.53 | 17.15 | 1 |  | -19.18 | 18.1 | 1 |
| Women: ALDH2*1/*1 vs ALDH2*1/*2 | 1 | 20.08 | 20.72 | 1 |  | 22.8 | 21.35 | 1 |
| ALDH2*1/*1: Women vs Men | 2 | 30.96 | 13.2 | 0.126 |  | 28.56 | 15.17 | 0.377 |
| ALDH2*1/*2: Women vs Men | 2 | -11.66 | 18.62 | 1 |  | -13.42 | 19.54 | 1 |
| Men: ALDH2*1/*1 vs ALDH2*1/*2 | 2 | -84.88 | 17.16 | 0 |  | -81.11 | 18.09 | 0 |
| Women: ALDH2*1/*1 vs ALDH2*1/*2 | 2 | -42.26 | 20.75 | 0.26 |  | -39.13 | 21.37 | 0.414 |
| ALDH2*1/*1: Women vs Men | 3 | 30.96 | 13.2 | 0.126 |  | 28.56 | 15.17 | 0.377 |
| ALDH2*1/*2: Women vs Men | 3 | -11.66 | 18.62 | 1 |  | -13.42 | 19.54 | 1 |
| Men: ALDH2*1/*1 vs ALDH2*1/*2 | 3 | -90.98 | 17.26 | 0 |  | -89.49 | 18.18 | 0 |
| Women: ALDH2*1/*1 vs ALDH2*1/*2 | 3 | -48.36 | 20.77 | 0.127 |  | -47.51 | 21.39 | 0.167 |
| ALDH2*1/*1: Women vs Men | 4 | 30.96 | 13.2 | 0.126 |  | 28.56 | 15.17 | 0.377 |
| ALDH2*1/*2: Women vs Men | 4 | -11.66 | 18.62 | 1 |  | -13.42 | 19.54 | 1 |
| Men: ALDH2*1/*1 vs ALDH2*1/*2 | 4 | -62.31 | 17.08 | 0.002 |  | -60.38 | 18.03 | 0.006 |
| Women: ALDH2*1/*1 vs ALDH2*1/*2 | 4 | -19.7 | 20.68 | 1 |  | -18.4 | 21.31 | 1 |
| ALDH2*1/*1: Women vs Men | 5 | 30.96 | 13.2 | 0.126 |  | 28.56 | 15.17 | 0.377 |
| ALDH2*1/*2: Women vs Men | 5 | -11.66 | 18.62 | 1 |  | -13.42 | 19.54 | 1 |
| Men: ALDH2*1/*1 vs ALDH2*1/*2 | 5 | -41.1 | 17.15 | 0.104 |  | -38.39 | 18.1 | 0.211 |
| Women: ALDH2*1/*1 vs ALDH2*1/*2 | 5 | 1.52 | 20.72 | 1 |  | 3.59 | 21.35 | 1 |
| ALDH2*1/*1: Women vs Men | 6 | 30.96 | 13.2 | 0.126 |  | 28.56 | 15.17 | 0.377 |
| ALDH2*1/*2: Women vs Men | 6 | -11.66 | 18.62 | 1 |  | -13.42 | 19.54 | 1 |
| Men: ALDH2*1/*1 vs ALDH2*1/*2 | 6 | -38.91 | 17.15 | 0.146 |  | -36.91 | 18.1 | 0.257 |
| Women: ALDH2*1/*1 vs ALDH2*1/*2 | 6 | 3.71 | 20.72 | 1 |  | 5.07 | 21.35 | 1 |
| ALDH2*1/*1: Women vs Men | 7 | 30.96 | 13.2 | 0.126 |  | 28.56 | 15.17 | 0.377 |
| ALDH2*1/*2: Women vs Men | 7 | -11.66 | 18.62 | 1 |  | -13.42 | 19.54 | 1 |
| Men: ALDH2*1/*1 vs ALDH2*1/*2 | 7 | -30.7 | 17.08 | 0.442 |  | -28.8 | 18.03 | 0.671 |
| Women: ALDH2*1/*1 vs ALDH2*1/*2 | 7 | 11.92 | 20.68 | 1 |  | 13.18 | 21.31 | 1 |
| ALDH2*1/*1: Women vs Men | 8 | 30.96 | 13.2 | 0.126 |  | 28.56 | 15.17 | 0.377 |
| ALDH2*1/*2: Women vs Men | 8 | -11.66 | 18.62 | 1 |  | -13.42 | 19.54 | 1 |
| Men: ALDH2*1/*1 vs ALDH2*1/*2 | 8 | -25.44 | 17.08 | 0.827 |  | -23.19 | 18.03 | 1 |
| Women: ALDH2*1/*1 vs ALDH2*1/*2 | 8 | 17.18 | 20.68 | 1 |  | 18.79 | 21.31 | 1 |

Note: EMMs = Estimated marginal means; VCRT = visual choice reaction time, ms. * *P* values were corrected for multiple comparison using Bonferroni method.

**Table S13** The estimated differences in EMMs for DSST between different sex or genotype at each time point post-consumption.

| Comparison | Time (h) | Main analyses | | |  | Analyses with adjustment of  age and alcohol consumption history | | |
| --- | --- | --- | --- | --- | --- | --- | --- | --- |
|  |  | Diff in EMMs | SE | *P* |  | Diff in EMMs | SE | *P* |
| ALDH2*1/*1: Women vs Men | 1 | 0.71 | 3.38 | 1 |  | 3.39 | 3.93 | 1 |
| ALDH2*1/*2: Women vs Men | 1 | 0.71 | 3.38 | 1 |  | 3.39 | 3.93 | 1 |
| Men: ALDH2*1/*1 vs ALDH2*1/*2 | 1 | 2.22 | 3.77 | 1 |  | -0.94 | 4.04 | 1 |
| Women: ALDH2*1/*1 vs ALDH2*1/*2 | 1 | 2.22 | 3.77 | 1 |  | -0.94 | 4.04 | 1 |
| ALDH2*1/*1: Women vs Men | 2 | 0.71 | 3.38 | 1 |  | 3.39 | 3.93 | 1 |
| ALDH2*1/*2: Women vs Men | 2 | 0.71 | 3.38 | 1 |  | 3.39 | 3.93 | 1 |
| Men: ALDH2*1/*1 vs ALDH2*1/*2 | 2 | 3.71 | 3.78 | 1 |  | 0.66 | 4.05 | 1 |
| Women: ALDH2*1/*1 vs ALDH2*1/*2 | 2 | 3.71 | 3.78 | 1 |  | 0.66 | 4.05 | 1 |
| ALDH2*1/*1: Women vs Men | 3 | 0.71 | 3.38 | 1 |  | 3.39 | 3.93 | 1 |
| ALDH2*1/*2: Women vs Men | 3 | 0.71 | 3.38 | 1 |  | 3.39 | 3.93 | 1 |
| Men: ALDH2*1/*1 vs ALDH2*1/*2 | 3 | 4.55 | 3.77 | 1 |  | 1.37 | 4.05 | 1 |
| Women: ALDH2*1/*1 vs ALDH2*1/*2 | 3 | 4.55 | 3.77 | 1 |  | 1.37 | 4.05 | 1 |
| ALDH2*1/*1: Women vs Men | 4 | 0.71 | 3.38 | 1 |  | 3.39 | 3.93 | 1 |
| ALDH2*1/*2: Women vs Men | 4 | 0.71 | 3.38 | 1 |  | 3.39 | 3.93 | 1 |
| Men: ALDH2*1/*1 vs ALDH2*1/*2 | 4 | 2.9 | 3.75 | 1 |  | 0.04 | 4.02 | 1 |
| Women: ALDH2*1/*1 vs ALDH2*1/*2 | 4 | 2.9 | 3.75 | 1 |  | 0.04 | 4.02 | 1 |
| ALDH2*1/*1: Women vs Men | 5 | 0.71 | 3.38 | 1 |  | 3.39 | 3.93 | 1 |
| ALDH2*1/*2: Women vs Men | 5 | 0.71 | 3.38 | 1 |  | 3.39 | 3.93 | 1 |
| Men: ALDH2*1/*1 vs ALDH2*1/*2 | 5 | -1.26 | 3.75 | 1 |  | -4.41 | 4.02 | 1 |
| Women: ALDH2*1/*1 vs ALDH2*1/*2 | 5 | -1.26 | 3.75 | 1 |  | -4.41 | 4.02 | 1 |
| ALDH2*1/*1: Women vs Men | 6 | 0.71 | 3.38 | 1 |  | 3.39 | 3.93 | 1 |
| ALDH2*1/*2: Women vs Men | 6 | 0.71 | 3.38 | 1 |  | 3.39 | 3.93 | 1 |
| Men: ALDH2*1/*1 vs ALDH2*1/*2 | 6 | -3.35 | 3.75 | 1 |  | -6.39 | 4.02 | 0.686 |
| Women: ALDH2*1/*1 vs ALDH2*1/*2 | 6 | -3.35 | 3.75 | 1 |  | -6.39 | 4.02 | 0.686 |
| ALDH2*1/*1: Women vs Men | 7 | 0.71 | 3.38 | 1 |  | 3.39 | 3.93 | 1 |
| ALDH2*1/*2: Women vs Men | 7 | 0.71 | 3.38 | 1 |  | 3.39 | 3.93 | 1 |
| Men: ALDH2*1/*1 vs ALDH2*1/*2 | 7 | -1.34 | 3.75 | 1 |  | -4.09 | 4.02 | 1 |
| Women: ALDH2*1/*1 vs ALDH2*1/*2 | 7 | -1.34 | 3.75 | 1 |  | -4.09 | 4.02 | 1 |
| ALDH2*1/*1: Women vs Men | 8 | 0.71 | 3.38 | 1 |  | 3.39 | 3.93 | 1 |
| ALDH2*1/*2: Women vs Men | 8 | 0.71 | 3.38 | 1 |  | 3.39 | 3.93 | 1 |
| Men: ALDH2*1/*1 vs ALDH2*1/*2 | 8 | -3.05 | 3.76 | 1 |  | -6.19 | 4.04 | 0.766 |
| Women: ALDH2*1/*1 vs ALDH2*1/*2 | 8 | -3.05 | 3.76 | 1 |  | -6.19 | 4.04 | 0.766 |

Note: EMMs = Estimated marginal means; DSST = digit symbol substitution test. * *P* values were corrected for multiple comparison using Bonferroni method.

**Table S14** The estimated differences in EMMs for PTT between different sex or genotype at each time point post-consumption.

| Comparison | Time (h) | Main analyses | | |  | Analyses with adjustment of  age and alcohol consumption history | | |
| --- | --- | --- | --- | --- | --- | --- | --- | --- |
|  |  | Diff in EMMs | SE | *P* |  | Diff in EMMs | SE | *P* |
| ALDH2*1/*1: Women vs Men | 1 | -3.83 | 0.99 | 0.001 |  | -3.9 | 1.03 | 0.001 |
| ALDH2*1/*2: Women vs Men | 1 | -3.83 | 0.99 | 0.001 |  | -3.9 | 1.03 | 0.001 |
| Men: ALDH2*1/*1 vs ALDH2*1/*2 | 1 | 0.99 | 0.73 | 1 |  | 1 | 0.76 | 1 |
| Women: ALDH2*1/*1 vs ALDH2*1/*2 | 1 | 0.99 | 0.73 | 1 |  | 1 | 0.76 | 1 |
| ALDH2*1/*1: Women vs Men | 2 | -3.11 | 0.99 | 0.012 |  | -3.17 | 1.03 | 0.016 |
| ALDH2*1/*2: Women vs Men | 2 | -3.11 | 0.99 | 0.012 |  | -3.17 | 1.03 | 0.016 |
| Men: ALDH2*1/*1 vs ALDH2*1/*2 | 2 | 2.06 | 0.73 | 0.032 |  | 2.05 | 0.76 | 0.048 |
| Women: ALDH2*1/*1 vs ALDH2*1/*2 | 2 | 2.06 | 0.73 | 0.032 |  | 2.05 | 0.76 | 0.048 |
| ALDH2*1/*1: Women vs Men | 3 | -1.96 | 0.99 | 0.294 |  | -2.04 | 1.03 | 0.3 |
| ALDH2*1/*2: Women vs Men | 3 | -1.96 | 0.99 | 0.294 |  | -2.04 | 1.03 | 0.3 |
| Men: ALDH2*1/*1 vs ALDH2*1/*2 | 3 | 1.08 | 0.73 | 0.843 |  | 1.1 | 0.76 | 0.907 |
| Women: ALDH2*1/*1 vs ALDH2*1/*2 | 3 | 1.08 | 0.73 | 0.843 |  | 1.1 | 0.76 | 0.907 |
| ALDH2*1/*1: Women vs Men | 4 | -2.12 | 0.99 | 0.198 |  | -2.23 | 1.03 | 0.193 |
| ALDH2*1/*2: Women vs Men | 4 | -2.12 | 0.99 | 0.198 |  | -2.23 | 1.03 | 0.193 |
| Men: ALDH2*1/*1 vs ALDH2*1/*2 | 4 | 0.11 | 0.71 | 1 |  | 0.16 | 0.74 | 1 |
| Women: ALDH2*1/*1 vs ALDH2*1/*2 | 4 | 0.11 | 0.71 | 1 |  | 0.16 | 0.74 | 1 |
| ALDH2*1/*1: Women vs Men | 5 | -0.52 | 0.99 | 1 |  | -0.66 | 1.03 | 1 |
| ALDH2*1/*2: Women vs Men | 5 | -0.52 | 0.99 | 1 |  | -0.66 | 1.03 | 1 |
| Men: ALDH2*1/*1 vs ALDH2*1/*2 | 5 | -0.65 | 0.72 | 1 |  | -0.55 | 0.75 | 1 |
| Women: ALDH2*1/*1 vs ALDH2*1/*2 | 5 | -0.65 | 0.72 | 1 |  | -0.55 | 0.75 | 1 |
| ALDH2*1/*1: Women vs Men | 6 | -0.52 | 0.99 | 1 |  | -0.62 | 1.03 | 1 |
| ALDH2*1/*2: Women vs Men | 6 | -0.52 | 0.99 | 1 |  | -0.62 | 1.03 | 1 |
| Men: ALDH2*1/*1 vs ALDH2*1/*2 | 6 | -0.47 | 0.71 | 1 |  | -0.43 | 0.74 | 1 |
| Women: ALDH2*1/*1 vs ALDH2*1/*2 | 6 | -0.47 | 0.71 | 1 |  | -0.43 | 0.74 | 1 |
| ALDH2*1/*1: Women vs Men | 7 | 0.38 | 0.99 | 1 |  | 0.23 | 1.03 | 1 |
| ALDH2*1/*2: Women vs Men | 7 | 0.38 | 0.99 | 1 |  | 0.23 | 1.03 | 1 |
| Men: ALDH2*1/*1 vs ALDH2*1/*2 | 7 | -0.73 | 0.71 | 1 |  | -0.62 | 0.74 | 1 |
| Women: ALDH2*1/*1 vs ALDH2*1/*2 | 7 | -0.73 | 0.71 | 1 |  | -0.62 | 0.74 | 1 |
| ALDH2*1/*1: Women vs Men | 8 | -0.4 | 0.99 | 1 |  | -0.56 | 1.03 | 1 |
| ALDH2*1/*2: Women vs Men | 8 | -0.4 | 0.99 | 1 |  | -0.56 | 1.03 | 1 |
| Men: ALDH2*1/*1 vs ALDH2*1/*2 | 8 | -0.41 | 0.71 | 1 |  | -0.28 | 0.74 | 1 |
| Women: ALDH2*1/*1 vs ALDH2*1/*2 | 8 | -0.41 | 0.71 | 1 |  | -0.28 | 0.74 | 1 |

Note: EMMs = Estimated marginal means; PTT = pursuit tracking task. * *P* values were corrected for multiple comparison using Bonferroni method.
