# Supplementary figures and images for: Sex- and ALDH2-dependent differences in alcohol metabolism and psychomotor performance: a study in Han Chinese adults after binge drinking
Source: Ann Med. 2025 Apr 28;57(1):2496798. doi: 10.1080/07853890.2025.2496798 (PMC12039403; doi:10.1080/07853890.2025.2496798)

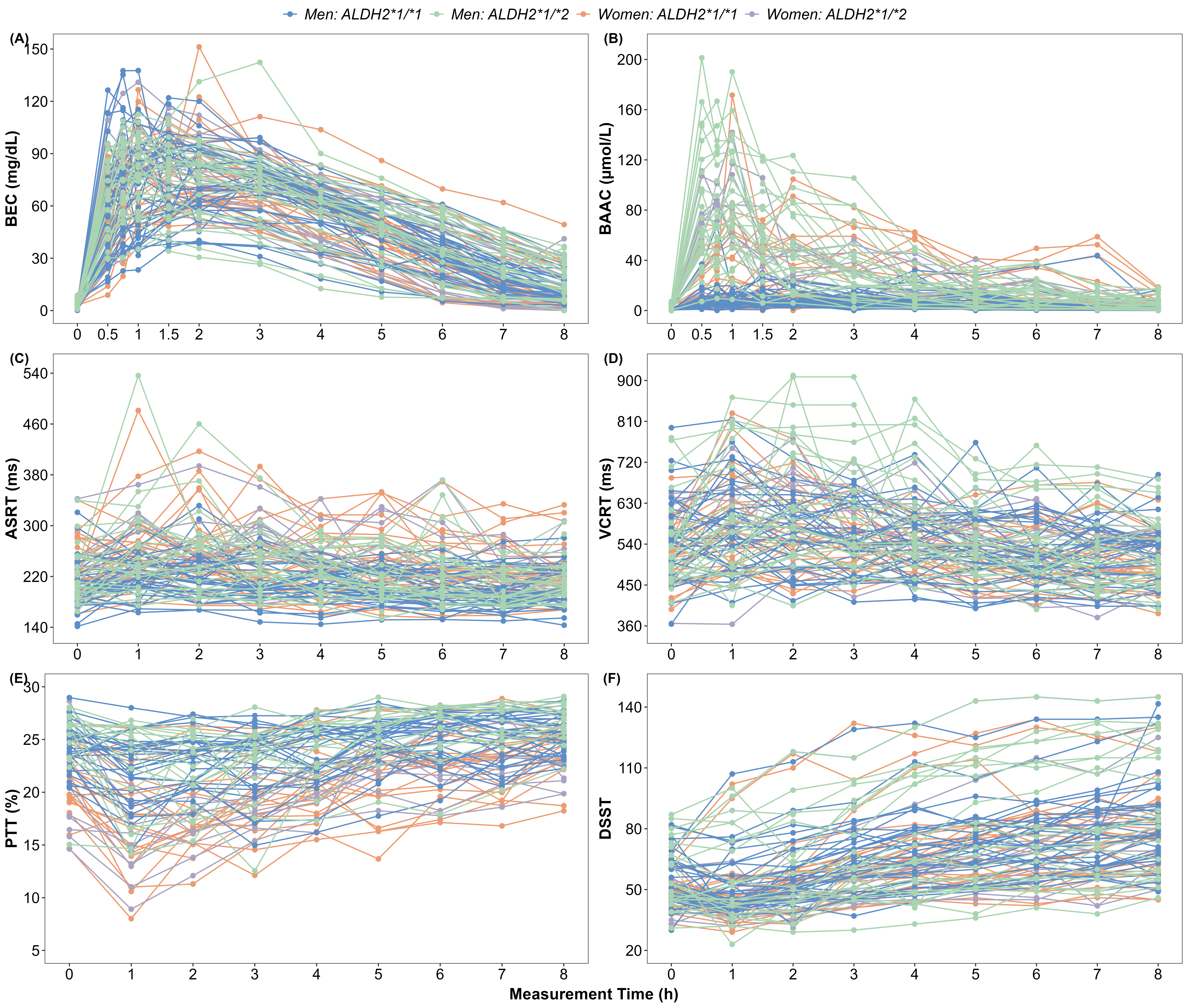

Supplement: Supplemental Material [file IANN_A_2496798_SM8626.zip › Sup/Supplementary Figure 1[AU].tif]

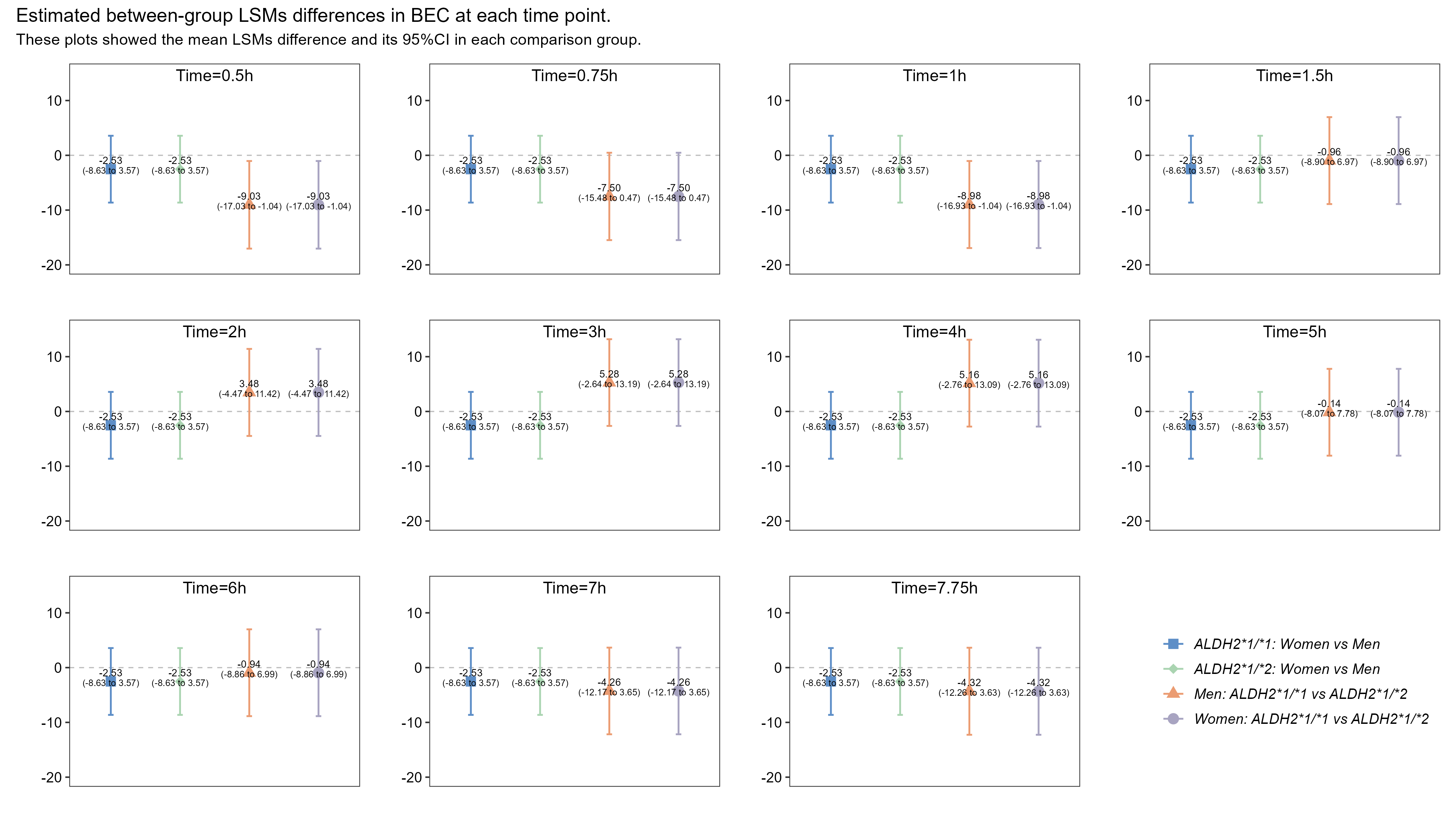

Supplement: Supplemental Material [file IANN_A_2496798_SM8626.zip › Sup/Supplementary Figure 2[AU].tif]

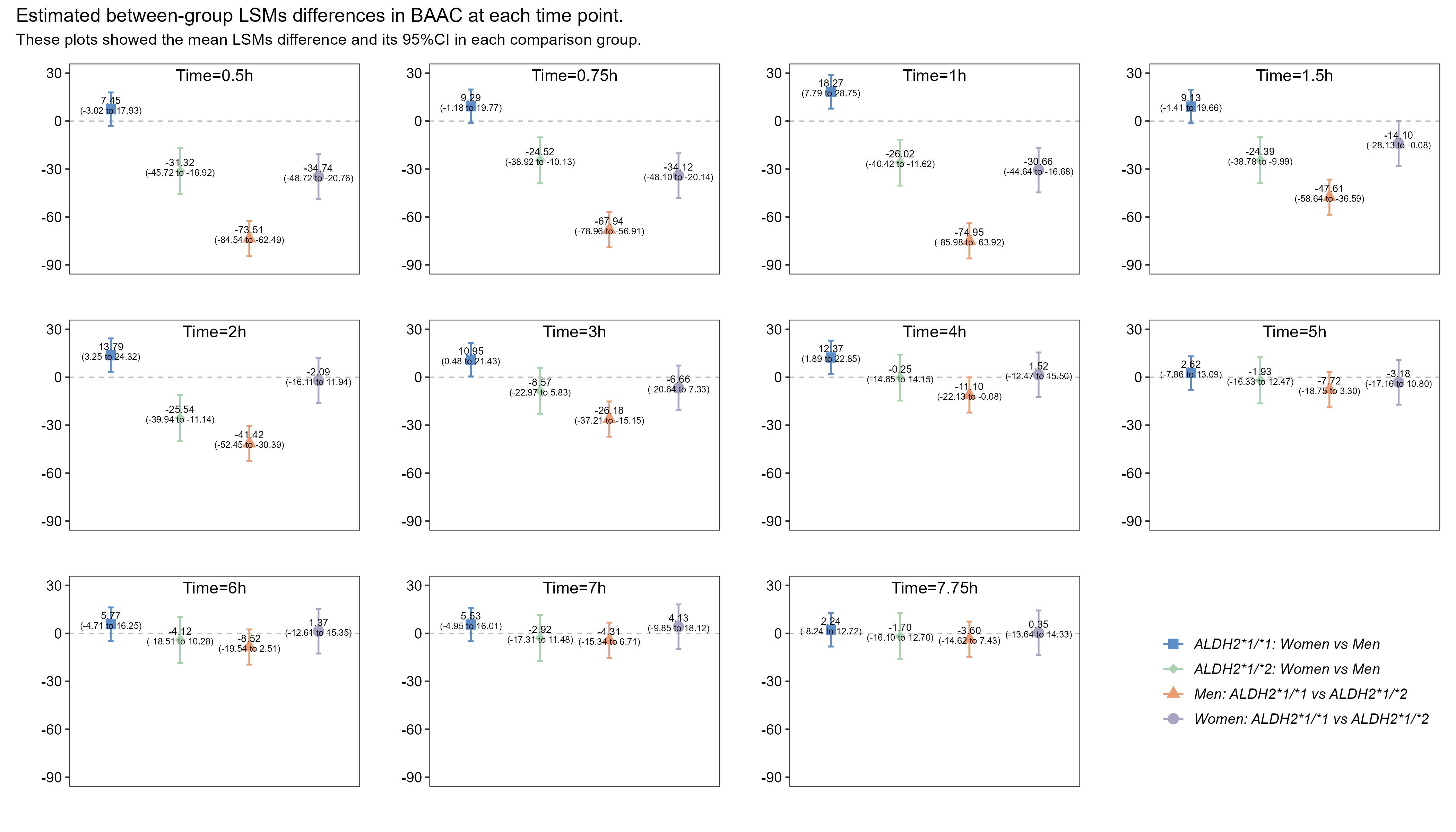

Supplement: Supplemental Material [file IANN_A_2496798_SM8626.zip › Sup/Supplementary Figure 3[AU].tif]

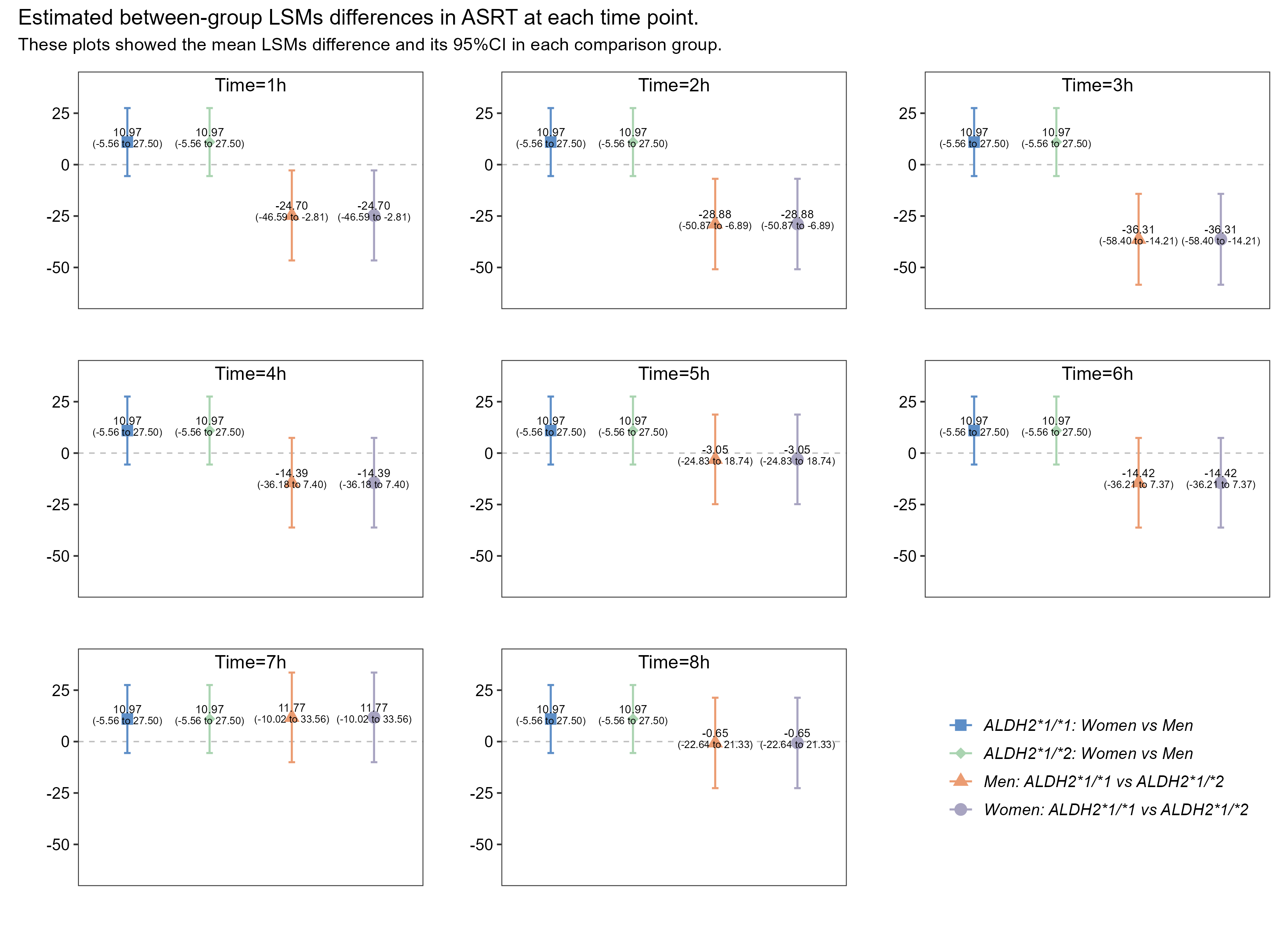

Supplement: Supplemental Material [file IANN_A_2496798_SM8626.zip › Sup/Supplementary Figure 4[AU].tif]

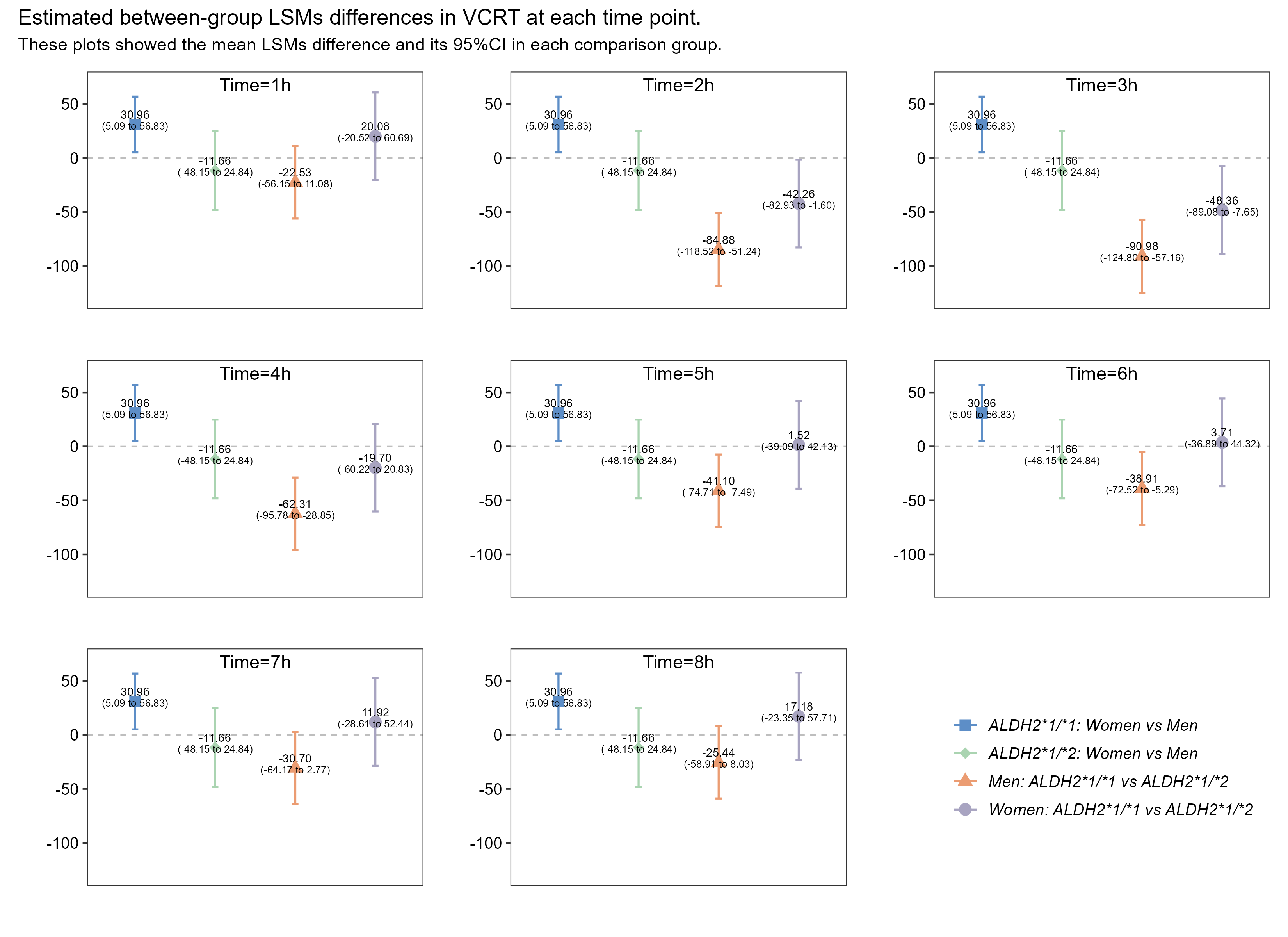

Supplement: Supplemental Material [file IANN_A_2496798_SM8626.zip › Sup/Supplementary Figure 5[AU].tif]

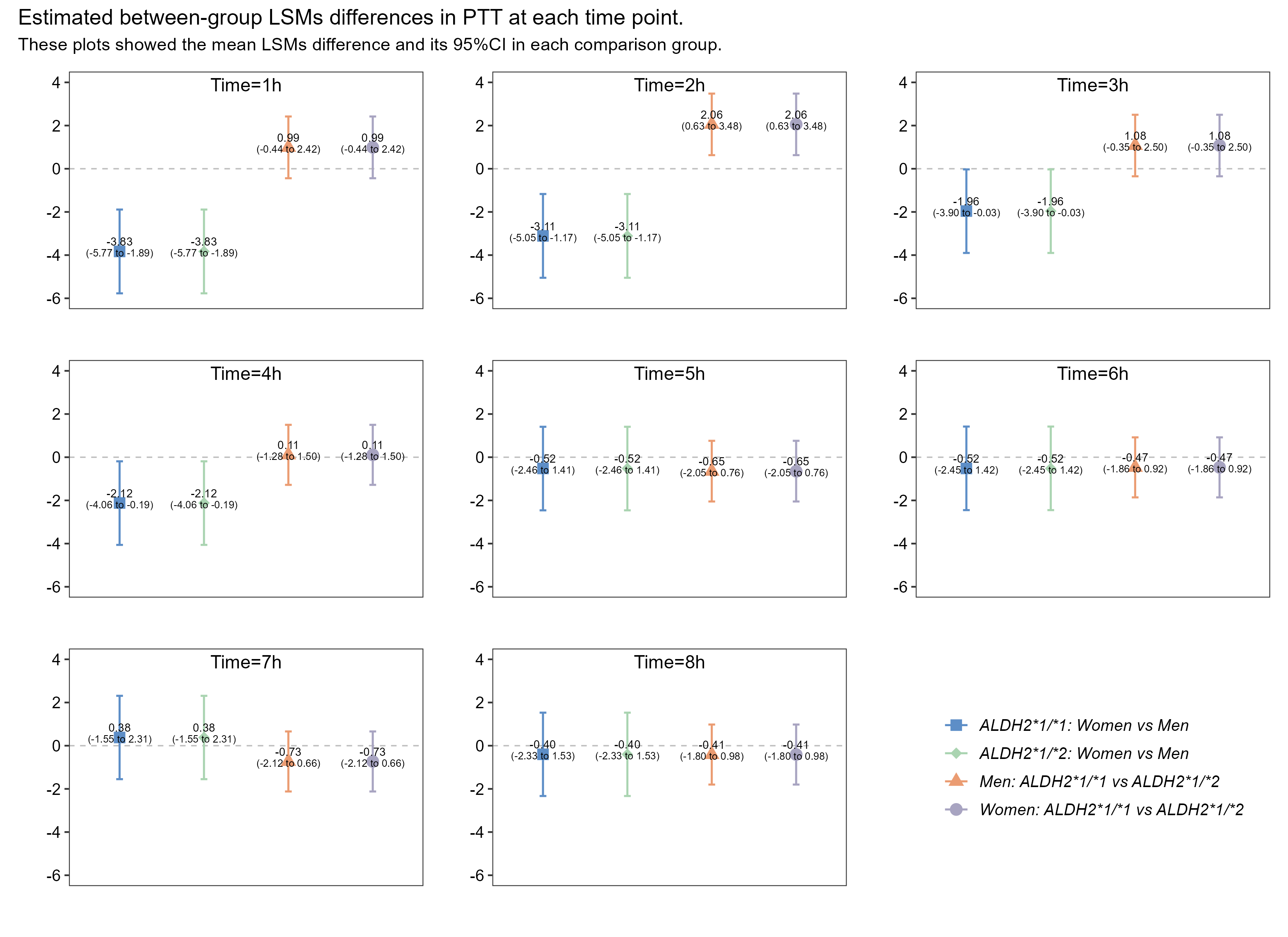

Supplement: Supplemental Material [file IANN_A_2496798_SM8626.zip › Sup/Supplementary Figure 6[AU].tif]

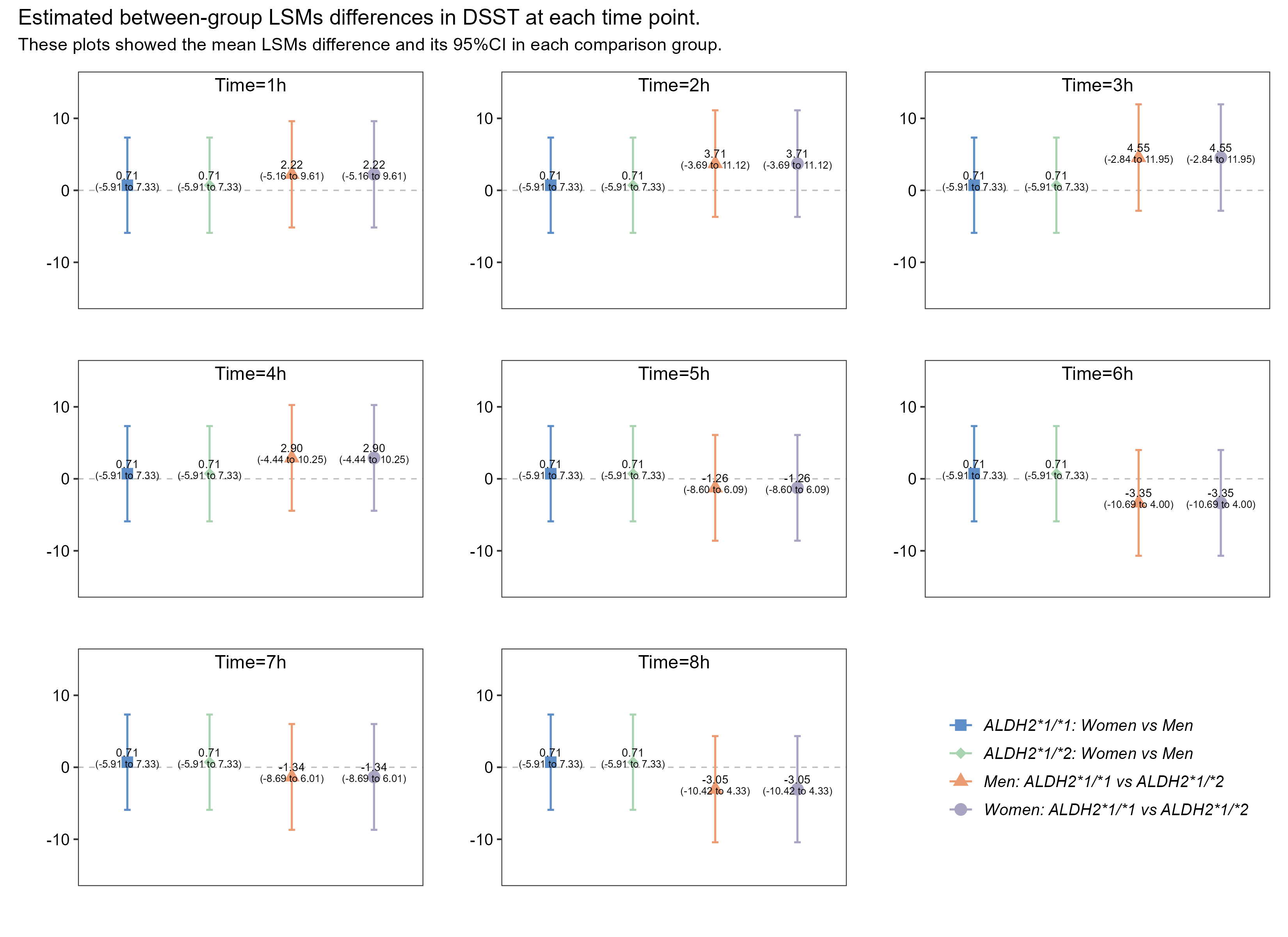

Supplement: Supplemental Material [file IANN_A_2496798_SM8626.zip › Sup/Supplementary Figure 7[AU].tif]
